# Detonation Nanodiamond Soot—A Structurally Tailorable Hybrid Graphite/Nanodiamond Carbon-Based Material

**DOI:** 10.3390/nano15010056

**Published:** 2025-01-01

**Authors:** Tikhon S. Kurkin, Oleg V. Lebedev, Evgeny K. Golubev, Andrey K. Gatin, Victoria V. Nepomnyashchikh, Valery Yu. Dolmatov, Alexander N. Ozerin

**Affiliations:** 1 Enikolopov Institute of Synthetic Polymer Materials Russian Academy of Sciences (ISPM RAS), Profsoyuznaya St. 70, 117393 Moscow, Russia; oleg.lebedev@phystech.su (O.V.L.); ozerin@ispm.ru (A.N.O.); 2 Moscow Center for Advanced Studies, Kulakova Str. 20, 123592 Moscow, Russia; 3 N. N. Semenov Federal Research Center for Chemical Physics Russian Academy of Sciences (FRCCP RAS), Kosygina Street 4, 119991 Moscow, Russia; 4 Federal State Unitary Enterprise, Special Design and Technology Bureau ‘Technolog’, Sovetsky Prosp. 33a, 192076 Saint Petersburg, Russia

**Keywords:** nanodiamond, nanodiamond soot, nanoparticles, graphite nanoribbons, polymer nanocomposites

## Abstract

The results of a comprehensive investigation into the structure and properties of nanodiamond soot (NDS), obtained from the detonation of various explosive precursors (trinitrotoluene, a trinitrotoluene/hexogen mixture, and tetryl), are presented. The colloidal behavior of the NDS particles in different liquid media was studied. The results of the scanning electron microscopy, dynamic light scattering, zeta potential measurements, and laser diffraction analysis suggested a similarity in the morphology of the NDS particle aggregates and agglomerates. The phase composition of the NDS nanoparticles was studied using X-ray diffraction, Raman spectroscopy, electron diffraction, transmission electron microscopy, atomic force microscopy, and scanning tunneling microscopy. The NDS particles were found to comprise both diamond and graphite phases. The ratio of diamond to graphite phase content varied depending on the NDS explosive precursor, while the graphite phase content had a significant impact on the electrical conductivity of NDS. The study of the mechanical and tribological characteristics of polymer nanocomposites, modified with the selected NDS particles, indicated that NDS of various types can serve as a viable set of model nanofillers.

## 1. Introduction

Hybrid carbon-based nanomaterials (HCNMs) have gained significant attention in a number of research areas and for a variety of practical applications [1,2,3,4]. HCNMs are defined as nanomaterials that combine different nanocarbon allotropes, offering a high degree of structural and morphological versatility [5]. This can result in a synergistic enhancement of their thermal, optical, or electrochemical properties while also improving their biocompatibility and processibility. As a result, the number of instances in which HCNMs are used as standalone engineered materials [4], as components of functional nanocomposite structures (e.g., polymer nanocomposites) [6], or as reinforced ceramics [3] is on the rise. However, these applications imply two major requirements for HCNMs and HCNM-based nanocomposite materials: (1) the fabrication method must be controllable, repeatable, and scalable, and (2) extensive characterization of HCNMs is necessary to enhance material design procedures [6]. Among the vast variety of HCNMs, two meet the first requirement: detonation nanodiamond (ND) and its precursor, detonation nanodiamond soot (NDS).

ND has been regarded as one of the most promising constituents for the design of nanocomposite materials and nanofluids over the past few decades [7,8,9,10]. The structure and morphology of ND particles have already been extensively investigated in a multitude of works [10,11,12]. For example, Shames et al. showed that ND defects are localized on the surface of diamond clusters [13]. Overall, ND particles retain the advantageous bulk characteristics of diamond, such as high hardness, chemical and bio-stability, thermal conductivity, etc. ND can be obtained industrially by detonating strong explosives of various types with subsequent purification of resulting carbon allotropes [8,14].

In the review by Shrestha et al. [15], it was concluded that chemical surface modification is an effective method for controlling the size of ND aggregates/agglomerates. Pastrana-Martínez et al. demonstrated the exceptional photocatalytic performance of functionalized ND particles for the degradation of a range of pharmaceuticals [16]. In their review [17], Ivanov et al. discussed the instances of successful implementation of detonation ND in nanolubricants and motor oils. Simioni et al. showed the potential of enhancing electrochemical sensors through ND particle modification, illustrating the versatility of ND for diverse biosensing applications [18].

The functional properties of ND allow for the effective enhancement of polymer characteristics, as evidenced by research in the works [19,20,21]. Consequently, ND-filled polymer nanocomposites have been a subject of intense research interest [22]. As illustrated in the review by Karami et al. [23], the exceptional properties of ND, coupled with its diverse surface functionality, make this type of carbon-based material an ideal filler for multifunctional polymer materials. Kidalov et al. [24] reported that the thermal conductivity of polymer nanocomposites modified with ND can reach as high as 10 W/(m∙K). Houshyar et al. successfully processed filaments based on polypropylene (PP) filled with ND particles using melt mixing [25]. The resulting filaments exhibited exceptional mechanical and biological properties as well as long-term stability. Furthermore, Kurkin et al. found that the addition of ND particles can significantly improve the adhesive strength of oriented poly(vinyl alcohol) (PVA) fibers in an epoxy binder, especially when both the polymer fiber and the cured epoxy matrix are filled with ND [20]. Additionally, Neitzel et al. [26,27] demonstrated that adding ND to an epoxy matrix provides an efficient route to decrease the friction coefficient of the resulting material. Nunes-Pereira et al. successfully prepared polymer nanocomposites with ND for tissue engineering applications, showing that ND can be used to tune the thermal and dielectric response of the polymer without affecting cell number and morphology in the composite samples [28].

Unfortunately, due to the complex purification process required to remove non-diamond carbon allotropes, using ND for modifying polymers can be cost-inefficient [9]. This is particularly relevant when high filler contents are needed to achieve the desired functional characteristics of a polymer nanocomposite. Furthermore, some important properties, such as electrical conductivity, cannot be effectively enhanced by incorporating ND into a polymer matrix [9].

Detonation nanodiamond soot (NDS), a precursor for ND, offers a more cost-effective alternative to ND while retaining some of its characteristics [22,29]. NDS is obtained as a direct post-detonation product of the ND synthesis, preceding the costly final stage of ND purification from the non-diamond carbon [14]. During the detonation synthesis of ND, the explosive precursor is subjected to a controlled detonation process. As the shockwave propagates, it leaves behind an aerosol of condensed carbon, which, after the removal of the metal residues, is considered to be NDS. While the conditions for detonation synthesis are often optimized for NDS to be enriched with the diamond phase [9,22], NDS commonly contains a range of fused carbon allotropes and non-carbon residues, which depend strongly on the type of explosives employed in the initial detonation. The majority of non-diamond carbon allotropes are located on the surface of the crystalline diamond cores. The latest results obtained by Dolmatov et al. indicate that the amount of diamond phase in NDS is affected not only by the detonation conditions (e.g., oxygen balance, reaction chamber geometry, the presence of additives, etc.) but also by the specific power of the explosive precursors and their mixtures [30]. In light of these findings, Dolmatov et al. put forth a hypothesis suggesting that the yield of the diamond phase during detonation synthesis can be predicted for a diverse array of explosive precursors [31]. It is therefore evident that the detonation synthesis precursors exert a direct influence on the balance of carbon allotropes in NDS. These findings offer a promising prospect for the development of NDS-based HCNMs with a diverse diamond/non-diamond phase composition ratio. NDS has the potential to contain a significant amount of non-diamond nanocarbon, such as nanographite, which can enhance its electrical conductivity. This allows for the effective use of NDS as a nanofiller in electrically conductive polymer nanocomposites [10] while retaining some of the characteristics associated with the presence of the crystalline diamond phase.

In order to effectively use NDS as a component for polymer nanocomposites, especially when aiming to combine the properties of diamond and non-diamond carbon allotropes in the resulting composites, it is necessary that the selected NDS powders adhere to the second principle for the design of HCNMs mentioned above. It is well known that the performance of nanofillers in polymer nanocomposites is generally related to the structural properties of the filler particles [32,33]. X-ray diffraction (XRD), dynamic light scattering (DLS), laser diffraction (LD), and a variety of microscopy techniques are the most commonly used methods for investigating the phase composition, structural hierarchy, and colloidal behavior of nanosized fillers. While each of these methods has inherent limitations, a combination of these methods often provides complementary information [32,33]. This information is crucial for successful optimization of nanocomposite material design and processing [34].

At present, a relatively limited number of studies have been published on the structure of NDS powders and polymers filled with NDS. For example, in the works of Satonkina et al. [10] and Chen et al. [29], the structure, composition, and surface properties of detonation NDS were characterized using a wide range of methods. These methods included high-resolution transmission electron microscopy (HR-TEM); Raman, Fourier transform infrared, and X-ray photoelectron spectroscopies; elemental analysis; XRD; and small-angle X-ray scattering. The selected NDS particles were found to possess a complex composition, comprising clusters of ND particles, nanographite, and nanosized amorphous carbon. Two distinct phases of graphite were observed in the NDS: mantles surrounding the ND cores, graphite nanospheres, and highly curved and entangled graphite nanoribbons. Alaferdov et al. demonstrated the effective use of such graphite nanoribbons in obtaining polymer composites based on ultra-high molecular weight polyethylene (UHMWPE) with a low percolation threshold (~0.42 vol.%) and a very high electrical conductivity (up to ~40 S/cm) [35].

The results of the investigation into the structure and characteristics of the NDS, obtained through detonation of 2,4,6-trinitrotoluene (TNT), led to the conclusion that a highly electrically conductive type of NDS can compete with such conventional nanosized fillers, such as carbon black (CB), for use in electrically conductive polymer nanocomposites [36]. Moreover, it was demonstrated that the presence of the diamond phase in the NDS powder enables the effective alteration of the mechanical characteristics of the polymer matrix.

In the study conducted by Kurkin et al. [37], oriented PVA fibers filled with detonation NDS were obtained and investigated using XRD, transmission electron microscopy (TEM), and mechanical testing. The findings indicated that the integration of NDS into the PVA matrix led to an improvement in the mechanical characteristics of the oriented PVA fibers. The maximum values of the elastic modulus and stored elastic energy were observed for the modified PVA fiber at relatively low NDS content values (~1 vol.%). Concurrently, the NDS particles were observed to retain a nanoscale size (less than 80 nm) within the PVA matrix, exhibiting no evidence of agglomeration up to a filling degree of 3 vol.%. Additionally, it was demonstrated that the adhesion strength between the epoxy matrix and the oriented NDS-filled PVA fibers was markedly superior to that observed for the non-modified fibers.

In the study conducted by Lebedev et al. [38], the percolation and tribological characteristics of PP-based nanocomposites filled with NDS particles were investigated. The corresponding results confirmed the formation of an NDS-enriched surface layer during annealing of the composites at temperatures above the melting point of PP. This effect was specifically addressed in the work [38] for electrically conductive fillers, such as CB, and attributed to the process of temperature-induced migration of the filler nanoparticles onto the surface of the nanocomposite material.

A review of the literature reveals that the majority of published papers focus on the study of ND powders or polymer nanocomposites modified with ND. There is a significant gap in the literature with regard to studies on the structure and properties of NDS obtained from various explosives and their mixtures. It is evident that further information is required regarding the structural and morphological characteristics of NDS with varying carbon phase compositions. This is crucial for the effective application of NDS as a highly versatile nanodispersed filler, which can be produced on an industrial scale in a controlled and consistent manner. Additionally, there can be potential benefits for numerical and theoretical modeling as well as for the development of semi-empirical models to study the relationship between composite structure, carbon filler type, and its content in a polymer matrix.

In this study, an in-depth comparative analysis was conducted to examine the structural and compositional characteristics of NDS obtained from a range of explosive precursors. The studied NDSs were characterized by a variety of analytical methods, including scanning electron microscopy (SEM), LD, TEM, XRD, Raman spectroscopy, electron diffraction analysis, atomic force microscopy (see Supplementary Materials), scanning tunneling microscopy (STM), and electrical conductivity measurements. The colloidal behavior of NDS nanoparticles in various liquid media was investigated using dynamic light-scattering (DLS) and zeta potential measurements. To provide insight into the potential for modeling with the selected set of NDS particles, an investigation was conducted into the tribological and mechanical properties of polymer nanocomposites based on the polymers, thoroughly investigated in our previous works (PP [38,39,40] and disentangled UHMWPE [39,41]), and modified with the selected NDS nanoparticles. The data obtained can serve as a foundation for future development of new HCNM-based polymer nanocomposites.

## 2. Materials and Methods

### 2.1. Materials

In this study, three distinct types of NDS were examined. The NDS powders were produced under different detonation synthesis conditions (Special Design and Technological Bureau “Technolog”, St. Petersburg, Russia). The “NDS-1” and “NDS-2” powders were obtained from TNT and a 1:1 mass ratio mixture of TNT and 1,3,5-trinitro-1,3,5-triazinane (hexogen), respectively. The “NDS-3” powder was obtained from 2,4,6-trinitrophenylmethylnitramine (tetryl), which is a novel and promising precursor in detonation synthesis applications. The corresponding synthesis procedures have been previously described in detail by Dolmatov et al. [9,22].

Commercial semicrystalline PP H030 GP/3 (SIBUR International GmbH, Vienna, Austria) was used as the matrix polymer for the nanocomposite materials to investigate the influence of the NDS type and content in the polymer matrix, as well as the effect of preliminary annealing at 205 °C, on the tribological properties of the nanocomposites.

Disentangled UHMWPE reactor powder (RP) was used for the solid-state processed nanocomposites modified with NDS. The UHMWPE RP was synthesized using a functionalized fluorine-substituted bis-(phenoxyimine) catalytic complex of titanium chloride (co-catalyst—methylaluminoxane at methylaluminoxane/Ti ratio = 500/1), the structure of which is described in detail by Ivanchev et al. [42].

### 2.2. Methods

SEM was employed to examine the NDS powders utilizing a JSM-6000 (Neoscope II) scanning microscope (Jeol, Tokyo, Japan).

In order to ascertain the hydrodynamic diameter and zeta potential values for the NDS nanoparticle agglomerates and aggregates in a variety of liquid media, including acetone, ethanol, dimethyl sulfoxide (DMSO), and isopropanol, a Zetatrac (Microtrac Inc., Montgomeryville, PA, USA) particle size and zeta potential analyzer was used. In a controlled reference self-beating mode, the scattered light was detected at 180° for the DLS measurements. The NDS agglomerate/aggregate size distributions were determined at room temperature by analyzing the power spectrum of a doppler-shifted signal from multiple trials. Each test sample was subjected to five 60-s measurement cycles. The sphere-equivalent hydrodynamic diameters of the NDS agglomerates/aggregates were subsequently calculated using the Stokes–Einstein equation, with solution viscosities for the liquid media at 25 °C. To determine the sign and magnitude of the zeta potentials, an alternating and direct current voltage was applied to the electrodes integrated into the sample cell. The zeta potential values were then calculated using the Smoluchowski equation from the electrophoretic mobility. The measurements and analysis were performed using the NanoFLEX software package. The NDS concentration employed in all the DLS and zeta potential measurements was 0.5 wt.%.

NDS agglomerate/aggregate size distributions in water were studied via LD using a Wintrac 3000 (Microtrac Inc., Montgomeryville, PA, USA) analyzer equipped with He-Ne (λ = 632.8 nm) and solid-state (λ = 405 nm) lasers. The NDS water dispersions were obtained via an ultrasound probe (60 W, 40 kHz) integrated into the circulation system. During the scattered intensity measurements, the samples were circulated through the measurement cell at a rate of 2000 rpm. For each sample, the results of 60 scans of 2 s each were obtained and averaged. The scattering patterns were then processed according to the Fraunhofer diffraction and Mie scattering models in order to obtain the particle size distributions.

The CUD-500 (Criamid, Moscow, Russia) ultrasonic disperser (250 W, 30 kHz) was employed for a two-minute treatment of the NDS dispersions prior to the DLS/LD/zeta potential measurements. During the interval between the conclusion of the dispersion process via ultrasonication and the commencement of the DLS/LD/zeta potential studies, the dispersions were maintained at room temperature in a sealed and dark environment.

TEM and electron diffraction studies were conducted using a fully digital 200 kV Tecnai Osiris (FEI Company, Hillsboro, OR, USA) scanning transmission electron microscope. Prior to the analysis, the samples were prepared by deposition of the NDS dispersions onto a holey carbon film on gold grids.

XRD diffractograms of the NDS powders were obtained using a D8 Advance (Bruker, Billerica, MA, USA) powder diffractometer (λ = 1.5418 Å, 2θ = 10–105°) equipped with a LYNXEYE 1D scintillating detector, focusing germanium crystal monochromator, and Ni-filter. XRD patterns were recorded in the transmission mode. The NDS powders were placed between two amorphous poly(ethylene terephthalate) films. The diffraction patterns were processed using the Origin Pro 2018b software package, which included peak separation, corrections for non-coherent background scattering, and scattering from the poly(ethylene terephthalate) films.

The NDS Raman spectra were obtained using a RamanStation-400 (Perkin-Elmer, Waltham, MA, USA) spectrometer. The spectrometer was equipped with a thermoelectrically cooled CCD matrix detector (Andor Inc., Belfast, North Ireland), a single-mode diode laser (785 nm, 1–100 mW), and a microscope lens (×30) for the collection of Raman photons and the delivery of laser excitation. The Raman spectra were obtained in a 180° backscattering configuration. The Raman spectra were obtained within the range of 3400–100 cm^−1^ Raman shifts at a resolution of 1 cm^−1^. The laser excitation power and signal accumulation time intervals utilized for the acquisition of the spectra were contingent upon the luminescence activity of the samples. For the various experiments, the laser excitation power ranged from 2 to 25 mW, with signal accumulation time intervals varying between 20 and 180 s. The dedicated Bruker OPUS 8.7 software package was employed for the routine processing of the Raman spectra, which included baseline correction and smoothing.

STM studies were conducted using a VT-STM (Omicron NanoTechnology, Taunusstein, Germany) scanning tunneling microscope at room temperature and a pressure of 2 × 10^−10^ Torr. In accordance with the established protocol, tungsten wire-based STM probes were utilized. The STM topography measurements were conducted in the constant current mode with a bias voltage of 2.0 V and a tunneling current of 3.1 nA. Prior to the STM investigations, NDS dispersions in isopropanol (with a solid phase concentration of 0.5 wt.%) were deposited on a highly oriented pyrolytic graphite substrate and allowed to dry in air at room temperature.

The DC electrical conductivity of the nanoparticle powders was determined using a 34401A (Agilent, Santa Clara, CA, USA) multimeter in the four-probe mode. To modify the volume fraction of the powders, a specialized apparatus was employed (see Section 3.8).

Tribological tests were conducted using a pin-on-disk testing machine (cylindrical stainless steel pin) in the dry friction mode, in accordance with the ASTM G99-05 standard, G99-05 (Reapproved 2016), ASTM International, West Conshohocken, PA, United States, 2016. The standard values of the test parameters are presented in Table 1. For each parameter set, the results of six identical test runs were obtained and averaged to determine the dependence of the friction coefficient on time. The final friction coefficient values were achieved through averaging the time dependencies of the friction coefficient in the range of 2–8 h, which encompasses the duration of the tribological test run. The relative humidity in the room was monitored and found to be within the range of 30–50%.

The Ez Test Ez-LX (Shimadzu, Kyoto, Japan) universal testing machine, equipped with a load cell up to 500 N, was used for mechanical testing of the UHMWPE-based nanocomposite tapes. The traverse speed was set to 2 mm/min until a pre-tension of 0.5 N was reached, after which the strain rate was set to 1 mm/min with the initial distance between the clamps of 100 mm. Tests were carried out until the specimen was ruptured. The tests were carried out until 3 reliable curves were obtained.

### 2.3. Nanocomposites Preparation

Samples of nanocomposites were obtained via melt mixing of the PP granules and NDS powders using a microcompounder (DACA Instruments, Goleta, CA, USA), which was designed to prepare compositions in laboratory quantities (up to 4 cm^3^). The quantity of material in the compounder chamber was calculated to ensure that the chamber was filled to capacity. The mixing procedure was conducted at 200 °C and 500 rpm for 10 min following the addition of the components into the compounder.

To prepare samples for tribological testing, granules of previously obtained PP-based nanocomposites of varying compositions were placed inside a heated, closed, circular mold with a height of 1 mm and a diameter of 7 cm. The composite granules were rapidly heated to 205 °C, followed by the gradual application of pressure (5 MPa). The pressure and temperature were maintained for a specified time period.

Nanocomposite mixtures for the solid-state processing (all processing stages occurring at temperatures below the melting point of the polymer) of the disentangled UHMWPE RP were obtained through simultaneous addition of the UHMWPE RP and NDS of a specific type in specified proportions in hexane, followed by ultrasonication treatment of the mixture in hexane for 10 min using the CUD-500 disperser. Ultrasonication treatment resulted in a fairly uniform distribution of the NDS on the surface of UHMWPE RP particles [41]. The resulting suspensions of UHMWPE RP particles coated with NDS particles were filtered out and dried until the dispersion medium was completely removed.

The solid-state processing of the UHMWPE RP and NDS mixtures was conducted in two stages. The initial stage involved compaction of the powdered mixture within a closed mold under a pressure of 300 MPa, resulting in the formation of rectangular samples with variable thicknesses, measuring 7 cm in length and 10 mm in width. At the second stage of processing, the rectangular samples were subjected to homogeneous shear deformation [43,44] by placing them into the clearance between two 155 mm diameter steel rollers heated to a temperature of 120 °C and rotating at a speed of 400 mm/min. The deformation ratio value was determined by calculating the ratio between the clearance width and the sample thickness.

## 3. Results and Discussion

The initial phase of the study involved an examination of the agglomeration and dispersion of NDS nanoparticles (see Section 3.1 and Section 3.2). This was done to obtain information about the conditions that favor the association of NDS nanoparticles.

The subsequent phase of the study involved an examination of the structural and phase composition of the individual nanoparticles (“primary particles”) and small hard aggregates of NDS of various types, along with their electrophysical characteristics (see Section 3.3, Section 3.4, Section 3.5, Section 3.6, Section 3.7 and Section 3.8). Such an investigation allowed us to understand the specifics and possible differences between the selected NDS nanoparticles. A model of structure of NDS particles of the selected types was suggested (see Section 3.9) based on the analysis of all the obtained experimental data.

The final phase of the study involved the fabrication of nanocomposite materials based on PP and UHMWPE and modified with various types of NDS (see Section 3.10). This illustrated the versatility of the selected set of NDS nanoparticles for possible practical applications.

### 3.1. SEM Results

**Figure 1:**
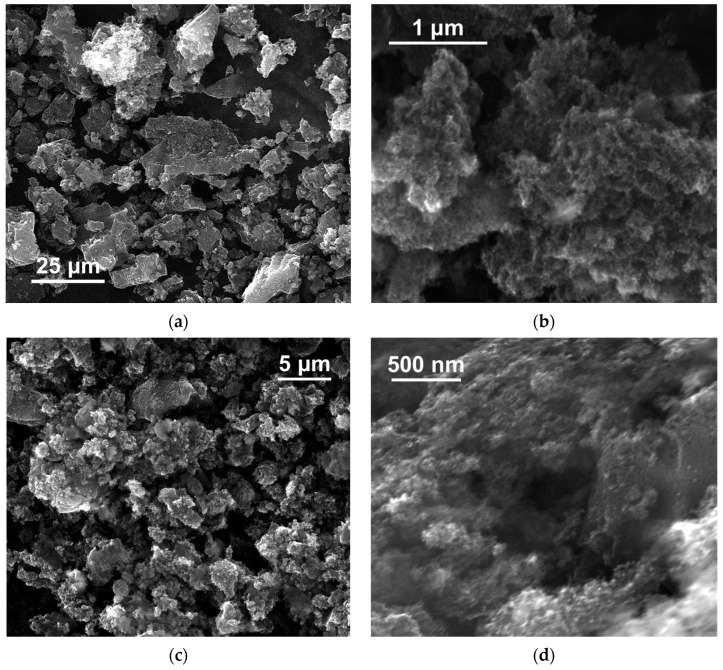

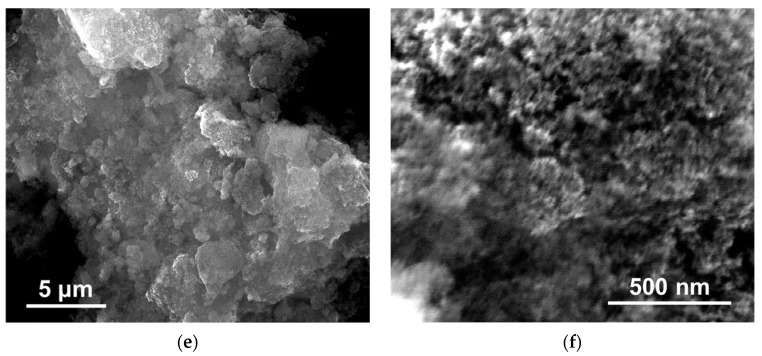
SEM images of the NDS powders: (**a**,**b**)—NDS-1; (**c**,**d**)—NDS-2; (**e**,**f**)—NDS-3.

The results of SEM studies of the powders of NDS of different types are presented in Figure 1. Additional SEM images of the NDS-1 powder can be found in the work [36].

It can be observed that, irrespective of the NDS type, the NDS powders contained agglomerates that were characterized by loose packaging of the carbon phase and an estimated size of >3 µm (Figure 1a,c,e). As observed, these relatively large agglomerates consisted of smaller aggregates of varying sizes (Figure 1b,d,f) [45]. The morphology of the NDS agglomerates can result from fluid removal during the final stages of the detonation synthesis [9,22]. Indeed, as the powders undergo the technological drying process, the primary NDS particles suspended in the reaction or purification liquid media undergo agglomeration, which can be distinctly observed in the SEM images. Since all the selected NDS types were subjected to similar drying conditions, the resulting agglomerate shapes and morphology proved to be similar.

### 3.2. DLS, Zeta Potential Measurements, and LD Results

**Figure 2:**
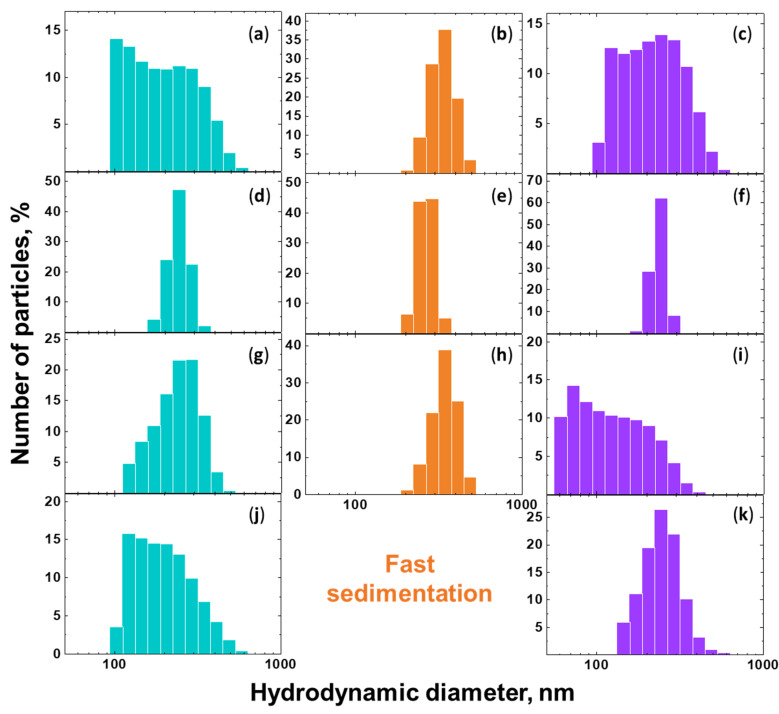
Distributions of hydrodynamic diameter of the (**a**,**d**,**g**,**j**) NDS-1, (**b**,**e**,**h**) NDS-2, and (**c**,**f**,**i**,**k**) NDS-3 nanoparticles in (**a**–**c**) ethanol, (**d**–**f**) isopropanol, (**g**–**i**) DMSO, and (**j**,**k**) acetone, obtained using the DLS technique.

The results of the DLS studies of the NDS particles of various types dispersed in diverse liquid media (ethanol, isopropanol, DMSO, and acetone) are presented in Figure 2.

For all the studied liquid media except water, the NDS aggregate/agglomerate size distributions were characterized by a median size of ~250 nm. The NDS-2 demonstrated the narrowest aggregate/agglomerate size distributions for all the liquid media studied, with the exception of acetone (normalized molar transition energy 0.355 [46]), as illustrated in Figure 2b,e,h. It was not possible to obtain a stable dispersion of NDS-2 in acetone, with the full NDS sedimentation time not exceeding ~2 min (referred to as “fast sedimentation” in Figure 2).

The median aggregate/agglomerate size for NDS-1, NDS-2, and NDS-3 was observed to be equal in the DLS data for isopropanol (normalized molar transition energy 0.546 [46]) used as the liquid medium (Figure 2d–f). Moreover, the NDS aggregate/agglomerate size distributions for all the NDS samples remained monomodal and narrow after one week (see Supplementary Materials). This suggests that not only the colloidal stability is the same for NDS-1, NDS-2, and NDS-3 in isopropanol but also that the conditions required for nanoparticle deagglomeration are similar for these powders in this particular liquid medium. Conversely, the NDS particle dispersions in water (normalized molar transition energy 1 [46]) were proven to be unstable for all the NDS types, not allowing for conduct size distribution studies using DLS. The aggregate/agglomerate size distributions obtained for the different types of NDS using ethanol (Figure 2a–c) and DMSO (Figure 2g–i) as the liquid media (normalized molar transition energies 0.654 and 0.444, respectively [46]) could be considered similar when comparing the results for the same NDS type.

NDS aggregate/agglomerate size distributions were obtained for varying storage times following ultrasonication. The samples were stored for 0 h, 24 h, 120 h, 1 week, 3 weeks, and 4 weeks prior to the DLS measurements. The results of the DLS measurements for different liquid media and storage times are presented in the Supplementary Materials. The distributions obtained were found to be similar to those obtained for NDS dispersions immediately after ultrasonication. The data obtained indicated that, irrespective of the conditions governing the detonation synthesis, the manner of self-organization of the selected NDS primary particles exhibited both macroscopic and microscopic morphological similarity. The polarity of the liquid medium determines the narrowness of the aggregate/agglomerate size distribution for the NDS powders, with the optimal polarity parameter (normalized molar transition energy) value being around ~0.55. While the NDS-2 demonstrated the best dispersions in most of the liquid media, its sedimentation rate was also proven to be the most sensitive to the environment.

**Figure 3:**
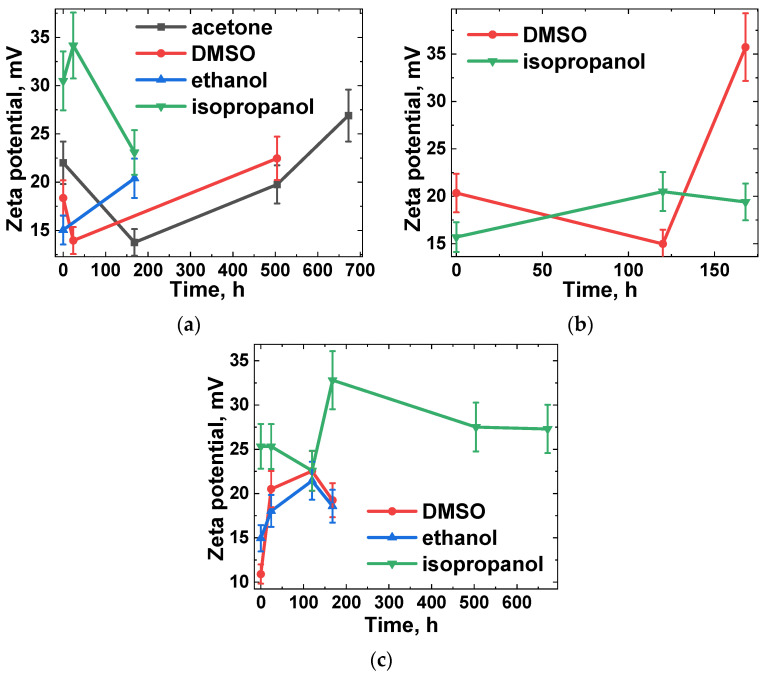
Results of zeta potential measurements for (**a**) NDS-1, (**b**) NDS-2, and (**c**) NDS-3 nanoparticles dispersed in different media versus the time between the ultrasonication procedure and the measurement of zeta potential of the NDS aggregates/agglomerates in the dispersions.

To further examine the stability of the NDS dispersions, the electrophoretic mobility of the NDS of all selected types was investigated in a range of liquid media and over varying time intervals between the initial ultrasonication and the measurements. The zeta potential values for the NDS dispersions in different media (ethanol, isopropanol, DMSO, and acetone) were obtained at 0 h, 24 h, 120 h, 1 week, 3 weeks, and 4 weeks after the initial ultrasonication. The results of the zeta potential measurements are presented in Figure 3.

As illustrated in Figure 3, the measured zeta potential values for all NDS types and liquid media were positive and exceeded 15 mV, with the exception of the measurements for the NDS-3 dispersion in DMSO immediately following ultrasonication. According to Ginés et al. [47], the positive zeta potentials observed for NDS dispersions can be attributed to the presence of the nanographite phase at the surface of the ND particles. Furthermore, it has been demonstrated that, contingent on NDS aggregate/agglomerate sizes, the dispersions can retain their size distributions for an extended duration if the corresponding zeta potentials are within the range of 20–30 mV (irrespective of the positive or negative zeta potential sign) [48,49].

The zeta potential values for NDS-1 dispersions remained within the range of uncertainty for all the investigated media, even after a four-week period: ~30 ± 5 mV for the dispersions in isopropanol, ~21 ± 5 mV for the dispersions in acetone, ~18 ± 4 mV for the dispersions in DMSO, and ~18 ± 4 mV for the dispersions in ethanol (Figure 3a). Due to the higher sedimentation rate of NDS-2 in different media, reliable results were only obtained for the dispersions of NDS-2 in DMSO and isopropanol (Figure 3b). For up until one week following the ultrasonication, the zeta potential of NDS-2 dispersions in both liquid media remained at ~18.5 ± 2.5 mV, with the exception of the zeta potential value for NDS-2 in DMSO after one week (~36 ± 4 mV). Similarly, no reliable results could be obtained for NDS-3 in acetone. However, the NDS-3 dispersions in isopropanol were found to be the most stable over a four-week period, with zeta potential values of ~27 ± 3 mV (Figure 3c). The zeta potential values for NDS-2 dispersions in DMSO and ethanol were observed to be ~18 ± 5 mV and ~18 ± 3 mV, respectively, for up to one week.

**Figure 4:**
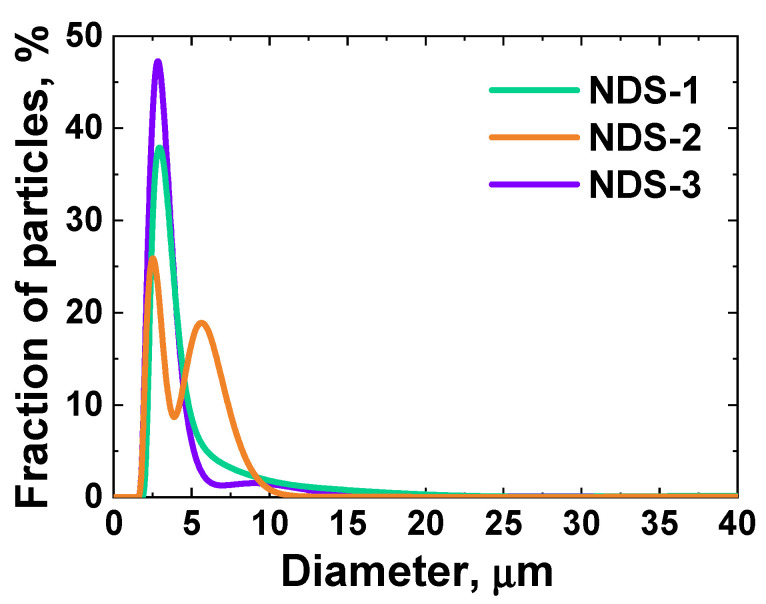
Distributions of diameter of the NDS particles in a suspension of NDS powders in water, obtained with the LD method for all the investigated NDS powders.

To examine the dispersion of NDS powders in a less compatible medium, such as water, the LD method was employed. This was made possible by the presence of a liquid circulation system with an integrated ultrasound probe in the measurement instrument. LD also allowed to take into account large NDS nanoparticle agglomerates, which were not detected by the DLS with sufficient accuracy due to the intrinsic limitations of the DLS technique. The results of the LD studies of the NDS powders of different types dispersed in water are presented in Figure 4.

As can be seen in Figure 4, the size distributions of the aggregates/agglomerates for all three NDS variants exhibited a peak maximum value of ~2.7 µm. In particular, the NDS water dispersion size distributions exhibited a peak value of ~2.9 µm for the NDS-1, ~2.5 and ~5.6 µm for the NDS-2, and ~2.8 µm for the NDS-3. The appearance of a second peak for NDS-2 at the higher size value of ~5.6 µm lends further support to the previous observations on the sensitivity of the NDS-2 dispersion to the polarity of the liquid media.

The absence of peaks at the low size values (<1 µm), as determined previously using the DLS method, can be attributed to the higher compatibility of the liquid media used for the DLS measurements as well as the pretreatment using an ultrasonic dispenser of higher power, in comparison to the dispenser used for the LD measurements. This enables the results obtained with LD to provide supplementary information regarding a distinct type of NDS agglomerate. As has been previously established in the works [15,45], the size of an aggregate or agglomerate is significantly influenced by the bonding strength between primary particles. This, in turn, determines the conditions necessary for disaggregation and deagglomeration. At the same time, agglomerates can be broadly classified as either “soft” or “hard” based on their bonding strength [45].

The results for the NDS aggregate/agglomerate size distributions in various liquid media obtained using DLS (Figure 2) and LD (Figure 4) exhibited some degree of agreement with the observations of NDS structures (primary particles, aggregates, and agglomerates [45]) made from the SEM data analysis for the NDS of various types (Figure 1). It can be surmised that under the conditions studied (media, characterized by the specific molar transition energy value), all NDS particles undergo deagglomeration from their initial agglomerated state (Figure 1) to an identical final state—nanoparticle aggregates with a diameter of ~250 nm.

It should be noted that the NDS aggregate/agglomerate size distributions obtained using DLS or LD methods are not necessarily indicative of the actual size distribution in a polymer nanocomposite modified with NDS nanoparticles. This is particularly relevant given that the nanoparticle aggregate/agglomerate size distribution can be readily influenced by the processing method employed in the production of the polymer nanocomposite [45]. However, it can be assumed that the fundamental characteristics and intrinsic morphology of the NDS primary particles (core composition, surface charge, and chemical composition) can determine certain structural hierarchies of the NDS particles, such as aggregates or “hard” agglomerates comprising relatively strongly bonded individual NDS particles, which can affect the processing routes during polymer nanocomposite design [15,45]. Therefore, the combination of DLS/LD techniques represents a valuable approach for obtaining a comprehensive understanding of the specifics of the macroscopic and microscopic structural organization of the NDS nanoparticles, depending on their explosive precursor.

### 3.3. TEM Results

**Figure 5:**
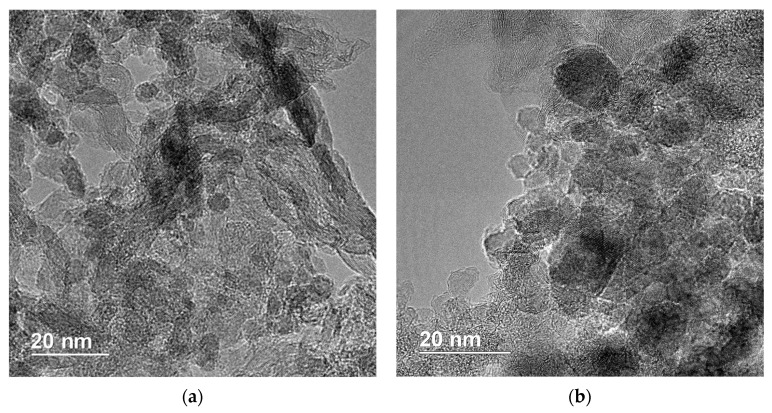

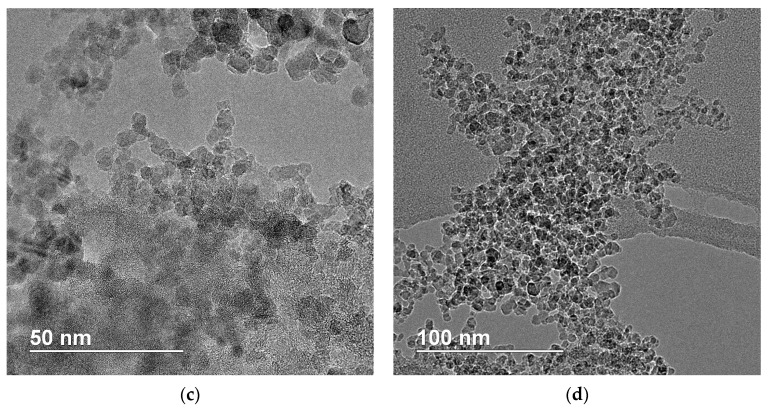
TEM images of the NDS powders: (**a**)—NDS-1; (**b**)—NDS-2; (**c**,**d**)—NDS-3.

The subsequent phase of the research involved an examination of the NDS primary particle structure. TEM was used as one of the main methods of NDS structure investigation. The corresponding results are presented in Figure 5. Additional TEM images of the NDS-1 powder can be found in the work [36].

The TEM images of the NDS-1 reveal the presence of graphite-enriched regions along with ND nanoparticles (Figure 5a). It can be observed that the graphite phase is partially manifested as graphite nanoribbons and nanobelts. The transverse dimensions of the nanoribbons can be roughly estimated as ~5–10 nm, while the high aspect ratio of the graphite nanoribbons is also evident. The relative ND content in NDS-2 (Figure 5b) is notably higher than in NDS-1 and is the highest in NDS-3 (Figure 5c) among all of the selected NDS types. In NDS-2, the boundaries of the ND particles are clearly discernible, while the graphite phase remains in abundance (Figure 5b). Ultimately, as the relative ND content of the NDS increases further, the diamond cores become larger and more densely packed (Figure 5c). The primary NDS particles, which are believed to be ND crystallites [23], appear to have an average diameter of ~5 nm. Additionally, nanoflakes of graphite (~10–20 nm in diameter) can be observed in NDS-2 and NDS-3 (Figure 5b,c). Low-magnification TEM images reveal the presence of larger dense aggregates (>200 nm) of primary NDS nanoparticles (Figure 5d). This observation is in agreement with the previously obtained results of SEM and DLS studies.

### 3.4. XRD Analysis Results

**Figure 6:**
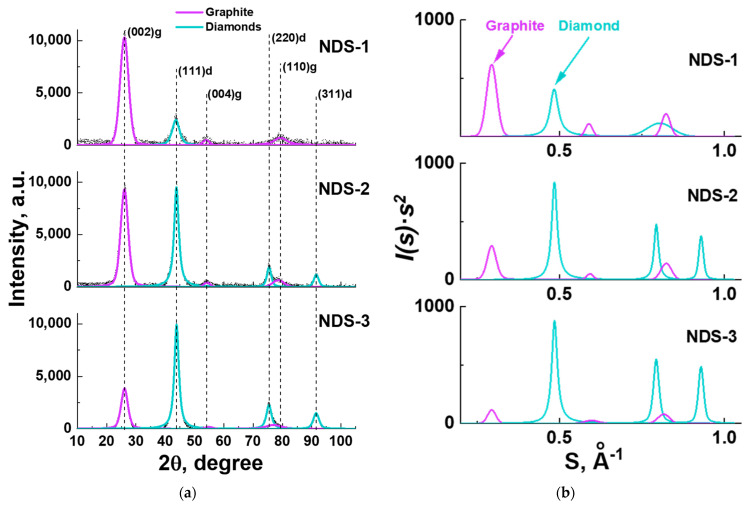
XRD patterns (CuK_α_-radiation) for various NDS types in (**a**) *I* − 2*θ* and in (**b**) *I*(*s*)*s*^2^*ds* − *s* coordinates. The peaks that are colored blue correspond to nanographite, while the peaks that are colored purple correspond to detonation nanodiamonds. Dashed vertical lines in (**a**) indicate the centers of the peaks.

To gain further insight into the structure and composition of NDS particles, the XRD method was employed. The results of the XRD analysis of the NDS powders of various types are presented in Figure 6.

From the obtained XRD patterns (Figure 6), the relative weight content of each carbon allotrope phase in NDS of various types was calculated. The XRD analysis entailed the deconvolution of the corresponding XRD diffractograms, thereby enabling the calculation of the contribution of each carbon allotrope to the scattering intensity. In order to determine the scattering coordinate, along with the scattering angle 2*θ* (Figure 6a), the value of the scattering vector modulus *s* = *s* = 4*π*sin(*θ*)/*λ* was used (Figure 6b), where *λ* = 0.1542 nm is the radiation wavelength. In such a case, integration must be performed over the backscattering sphere. This entails integrating not *I*(*s*)*ds* over all scattering angles but rather *I*(*s*)*s*^2^*d s*, which is known as Ruland’s method [50].

**Figure 7:**
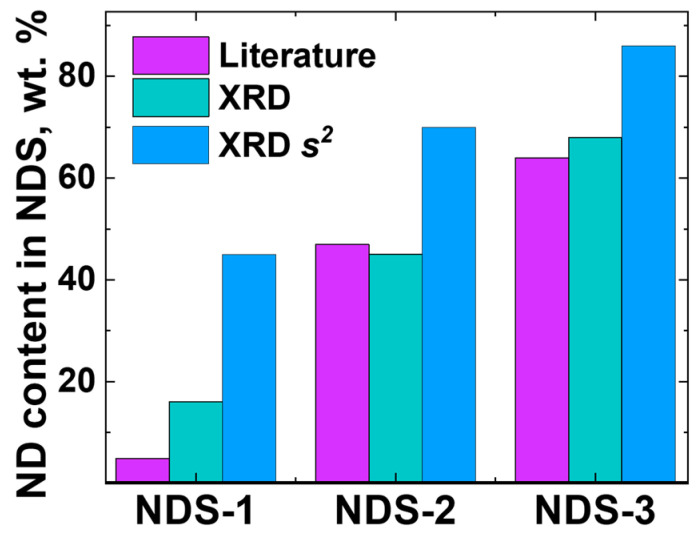
ND weight fraction values in the NDS powders of various types calculated using integration of the corresponding peaks in *I* − 2*θ* (“XRD”) and *I*(*s*)*s*^2^*ds* − *s* (“XRD s^2^”) coordinates. Literature data were taken from the publication of Dolmatov et al. [22].

It was determined that all three NDS powder types contained exclusively nanographite and ND phases. No non-carbon impurities were observed according to the XRD data. The results of the comparison of the relative ND content values for all the NDS powder types, obtained for both 2*θ* and s-coordinates, along with the literature data [22] are presented in Figure 7. The corresponding values of the ND and nanographite weight fraction values in the NDS powders of different types are provided in Table 2.

It is evident (Figure 7) that the ND phase content was in fact higher in NDS-2 in comparison to the ND content in NDS-1. The ND content of NDS-3 was found to be the highest among all the NDS types that were studied. This result was corroborated by the observations made during the analysis of the TEM data (Figure 5). As anticipated, the application of Ruland’s method resulted in an increase in the calculated ND content values compared to those obtained without its implementation. This was due to the higher scaling of *I*(*s*)*s*^2^ values at higher scattering vector modulus values compared to the intensity *I*. For the future calculations and references, the values of the ND fraction, calculated using integration of the corresponding peaks in *I* − 2*θ* coordinates, were used. Should the necessity arise, corrections to the data and graphs based on the ND content values were made using the data from Table 2.

The weight fraction values for the relative ND content in all the NDS powder types provided by the manufacturer [22] were found to be lower than the values obtained from the XRD analysis with and without the implementation of Ruland’s method (Figure 7). It can be reasonably assumed that the values obtained using the weighting procedure after the purification of ND may be lower than the actual ND content in the NDSs due to the potential for significant loss in the initial ND weight.

From the XRD data, it was possible to estimate the interlayer distance between the 002 planes of atoms in the nanographite phase. As indicated in the literature data [52], the ideal distance value is ~0.334 nm. The experimental value obtained from the XRD data (Figure 6) was found to be higher ((0.3413 ± 0.0003) nm) for all the investigated NDS powders. This suggests that the nanographite in all the NDS powder types was somewhat defective.

Furthermore, the XRD data analysis permitted the estimation of the nanographite coherently scattering domain size for the 002 reflection for all the examined NDS types through the application of the Scherrer equation [51]. The corresponding values of the domain sizes are presented in Table 2. The nanographite domain size was found to be the lowest for the NDS-1 and the highest for the NDS-3. This can be attributed to the different morphology of the graphite phase in the various types of NDS powders. For example, in the NDS-1 powder, the graphite phase is manifested in the form of graphite nanoribbons/nanobelts (Figure 5a), while in the NDS-3 powder, it is mostly observed covering the surface of the ND particles as mantles and relatively small graphite nanoflakes.

### 3.5. Raman Spectroscopy Results

**Figure 8:**
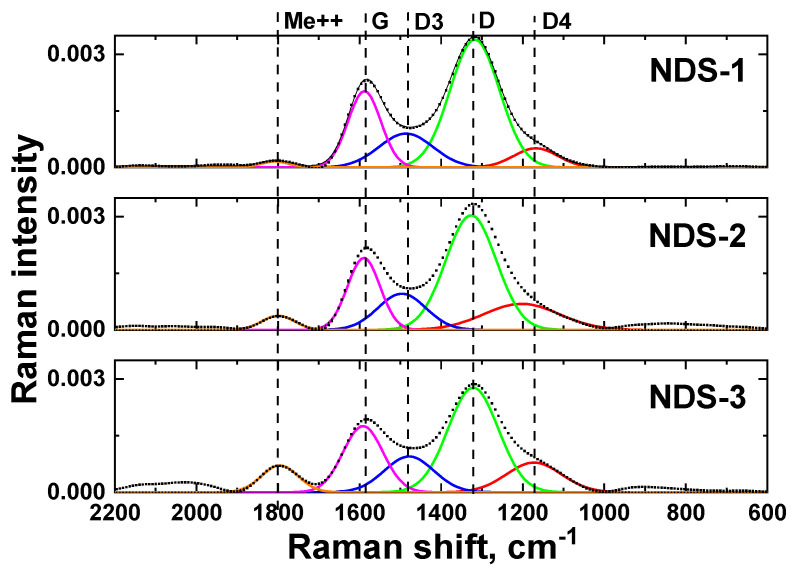
Raman spectroscopy results for NDSs of different types. Dotted lines represent the obtained experimental data, and solid lines are peaks from decomposition of the experimental data. Dashed vertical lines indicate the centers of the peaks.

In order to investigate the structure and composition of the nanographite phase in the investigated NDS powders, Raman spectroscopy measurements were performed on the selected NDS types. The results of the Raman spectroscopy measurements are shown in Figure 8.

As anticipated, the observed scattering can be attributed predominantly to nanographite particles (G and D bands). The main ND band is indistinct in the obtained Raman spectra due to the low ND nanoparticle sizes (<10 nm) and high nanographite D band intensity [53,54]. It is known that Raman scattering spectra of NDS cannot be described by G and D bands alone, as there are always two to three additional bands [31,32]. The Raman spectroscopy of the NDS powders studied in this work revealed two additional bands besides the commonly observed G and D bands. These additional bands are labelled as D3 and D4 in Figure 8, according to the literature [53,55]. The physical significance of these additional bands is discussed in the relevant publications [53,55,56]. Conventionally, the position of the D3 and D4 peaks is sensitive to various defects in the graphite phase. Since the peak positions were identical for all samples, it is reasonable to assume that the same form of nanographite was present in all the NDS types.

### 3.6. Electron Diffraction Analysis Results

**Figure 9:**
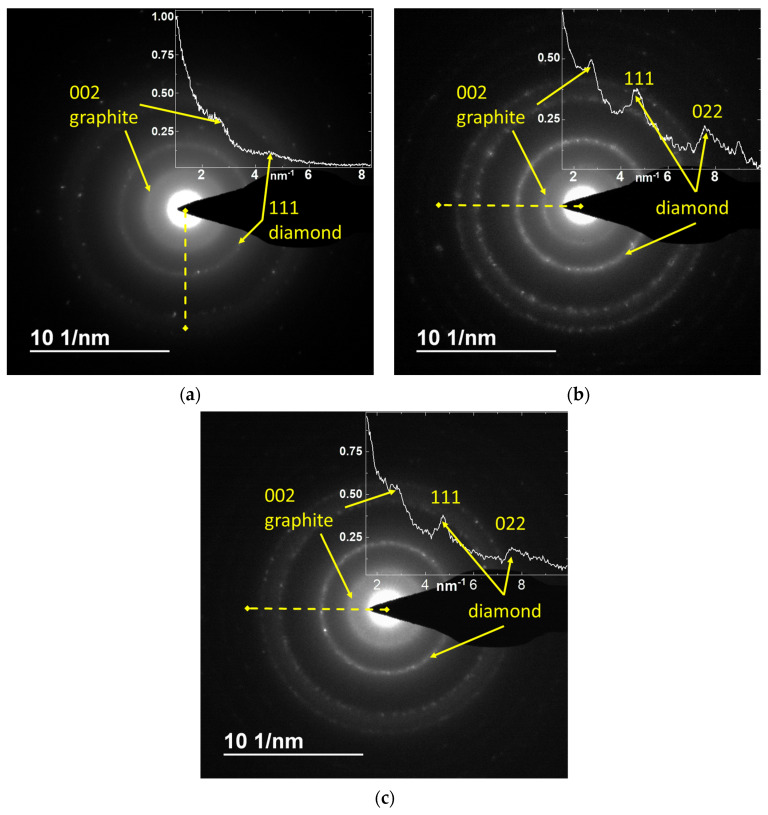
Electron diffraction results for the (**a**) NDS-1, (**b**) NDS-2, and (**c**) NDS-3. The insets in the diffractograms show distributions of normalized intensity along the dashed yellow lines. The yellow arrows point at the scattering peaks, corresponding to the diamond 111 and 022, and graphite 002 planes of carbon atoms.

In order to obtain additional data on the structure of the ND and nanographite phases in the selected NDSs, electron diffraction studies were carried out. The results of the electron diffraction analysis are shown in Figure 9.

The electron diffraction patterns (Figure 9) show characteristic reflections corresponding to the ND and nanographite phases for all the NDS particles. While the 022 diamond reflection was not distinguishable for the NDS-1 (Figure 9a), it is clearly visible in the diffraction patterns obtained for the NDS-2 (Figure 9b) and the NDS-3 (Figure 9c). From the analysis of the patterns obtained, the distance between the 002 graphite planes of the carbon atoms can be evaluated. The values obtained (3.45 ± 0.02 nm) were within the error of the value obtained experimentally from the XRD data (Figure 6a,b), further demonstrating the presence of defects in the nanographite phase for all the NDS particles.

### 3.7. STM Results

**Figure 10:**
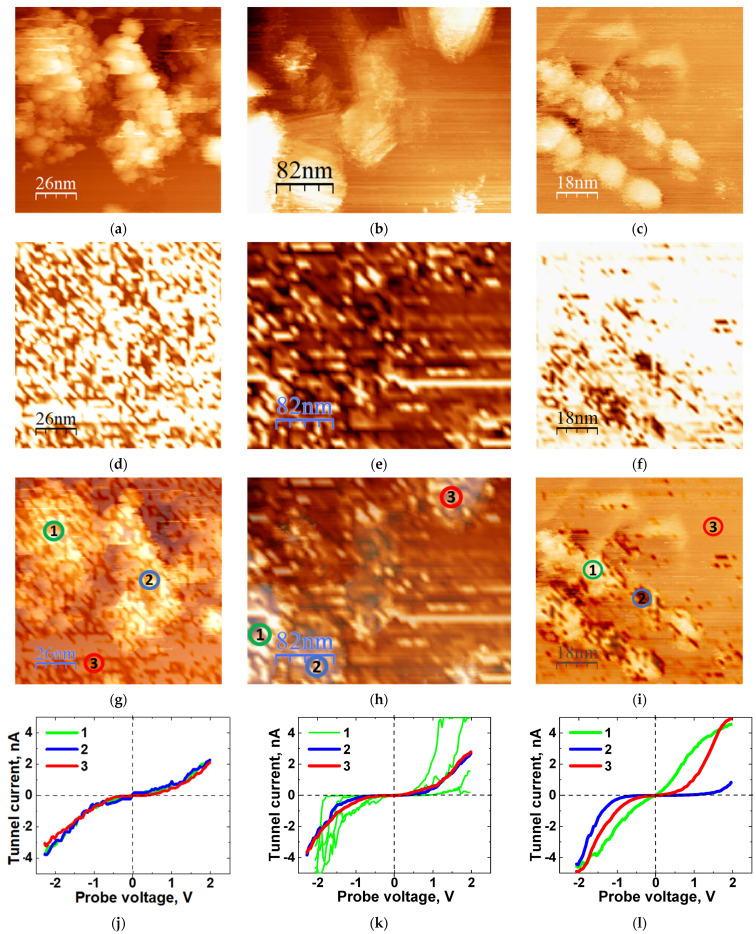
(**a**–**c**) Topography maps, (**d**–**f**) tunnel current distributions, (**g**–**i**) overlays of the corresponding tunnel current distributions with the topography maps, and (**j**–**l**) voltage-current curves obtained using the STM method for the (**a**,**d**,**g**,**j**) NDS-1, (**b**,**e**,**h**,**k**) NDS-2, and (**c**,**f**,**i**,**l**) NDS-3. The voltage-current curves correspond to the points indicated with the same number and color in the overlayed tunnel current maps (**g**–**i**).

Additional information on the structure and electrophysical properties of the NDS of various types was obtained using the STM technique. The results of the STM studies are presented in Figure 10.

The STM data enabled the visualization of isolated ND particles with a diameter of approximately 4–6 nm for all the NDS types under investigation (Figure 10a–c). This observation aligns with the findings derived from the TEM analysis (Figure 5). Furthermore, the presence of graphite nanoflakes is discernible, particularly in the case of the NDS-2 (Figure 10b).

The STM method also allowed us to obtain tunnel current maps for the NDS particles deposited on a highly oriented pyrolytic graphite substrate, thereby providing information on the local electrophysical characteristics of the NDS particles. Specifically, 2000 points were distributed uniformly across each image, and tunnel current/probe voltage curves were recorded for each point. The tunnel current values at a fixed voltage of +1 V were mapped as tunnel current distribution images across the same field of view as the topography images (Figure 10d–f). The tunnel current distributions were the most even for the NDS-1 (Figure 10d), while for NDS-3, the distributions were the least even (Figure 10f), especially in the vicinity of the NDS particle aggregates.

The tunnel current distributions were overlaid with the corresponding topography images, allowing for the selection of voltage-current curves for specific points of interest on the topography maps (Figure 10g–i). By analyzing the voltage-current curves for the graphite substrate and different regions of the NDS-1 particles, it was determined that the NDS-1 particles conduct electrical currents in a manner similar to that of the graphite substrate (Figure 10j). A similar analysis was conducted on the diamond-enriched NDS-3 powder. As previously stated, the NDS-3 aggregates exhibited strong negative contrast at various regions (Figure 10f,i). The voltage-current curves analysis demonstrated that these points are distinguished by a bandgap of ~1.5 V (Figure 10l), indicating that the material in these regions is semiconducting and can be attributed to the ND phase. For the NDS-2, which also exhibited uneven tunnel current distributions (Figure 10e,h), the voltage-current curves in the proximity of certain points, corresponding to the NDS-2 aggregates, demonstrated strongly fluctuating bandgap values. This is related to the composition of the NDS-2 primary particles, which is distinguished by the most balanced ND/nanographite content ratio among all the selected NDS types (Figure 7). Overall, the STM results demonstrate the versatility of the selected set of NDS types, allowing for a gradual transition of the electrophysical properties of the filler depending on the NDS synthesis method.

### 3.8. Electrical Conductivity Measurements

**Figure 11:**
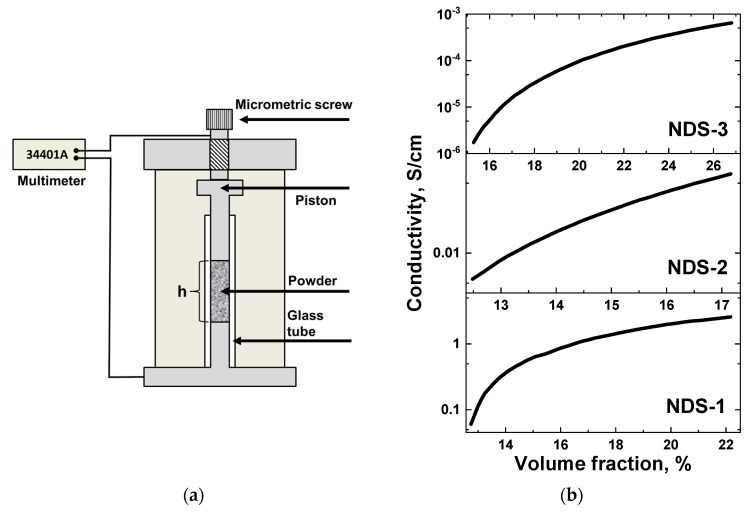
(**a**) Schematic image of the apparatus used for measuring specific resistivity of the NDS powders. (**b**) Conductivity of the NDS powders of various types versus volume fraction of the NDS nanoparticles in the measurement cell.

In order to assess the potential of the NDS as a nanofiller for enhancing the electrical conductivity of polymer materials, a specialized apparatus was employed to vary the volume fraction of the NDS powder while concurrently measuring the electrical resistance of the powder. A schematic representation of the aforementioned apparatus is provided in Figure 11a. In order to calculate the volume fraction of NDS, the densities of 1.94 and 3.3 g/cm^3^ were used for the ND and nanographite phases, respectively. The relative weight content of the phases in the NDS of various types was taken from the XRD analysis results (Table 2). The results of the electrical conductivity measurements for the compacted NDS powders versus the volume fraction of the NDS particles in the measurement cell are presented in Figure 11b.

As can be seen in Figure 11b, NDS-1 demonstrated the best electrical conductivity, comparable to that of the industrial electrically conductive CB. The NDS-2 powder showed a decrease of conductivity by an order of magnitude compared to NDS-1. In turn, the NDS-3 powder was characterized by conductivity of an order of magnitude lower than NDS-2.

As evidenced by the results of TEM (Figure 5), XRD (Figure 6), Raman spectroscopy (Figure 8), electron diffraction (Figure 9), and STM (Figure 10), a substantial quantity of nanographite (Figure 7) was present in all the NDS types, in conjunction with the NDs. The ND crystallites, with a diameter of ~4–5 nm, were surrounded by nanographite and assembled in relatively small, strongly coupled aggregates. Therefore, the high electrical conductivity of the NDS powders, particularly NDS-1, can be attributed to the presence of the nanographite phase in the form of nanoribbons. The high aspect ratio of graphite nanoribbons ensures very low percolation thresholds for electrical conductivity, as previously demonstrated by Alaferdov et al. [35]. These findings align with the results of a comparative study of the resistivity dependence on various volume fractions of NDS and CB powders [36] as well as with time-dependent resistivity measurements for composite melts containing NDS and CB [57].

### 3.9. NDS Nanoparticle Structure Representation

**Figure 12:**
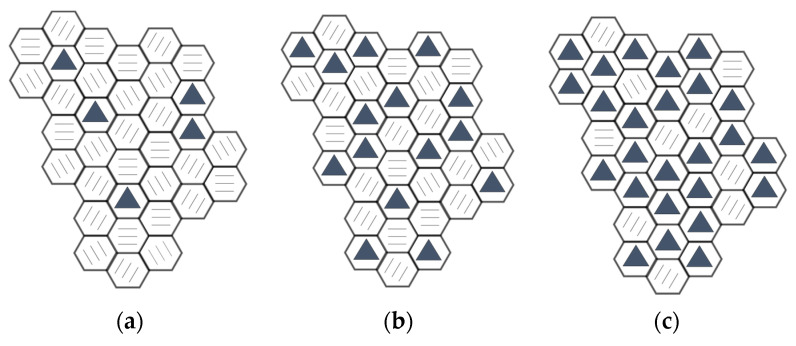
The assumed packaging of the ND (a hexagonal with a triangle inside) and nanographite (a hexagonal with three lines inside) subunits for the (**a**) NDS-1, (**b**) NDS-2, and (**c**) NDS-3 nanoparticles.

A summary of the data obtained from the structural investigations conducted revealed a certain degree of similarity in the structure of the NDS particles obtained under different conditions. The NDS particles, regardless of the explosive precursor, were found to consist of anisometric layers stacked upon each other at varying heights. Based on the aforementioned results, it appears that each layer consists of solely diamond and graphite, with the diamond content varying within a layer depending on the NDS type. The proposed packaging of the diamond and graphite subunits in NDS particles from different types of NDS is presented in Figure 12.

Remarkably, these conclusions correlate well with the small-angle X-ray scattering analysis of a different NDS type performed independently by Vornyakovskii et al. [58]. The reported model of an NDS nanoparticle was found to exhibit the shape of an oblate spheroid comprising non-diamond carbon enveloping ND cores, which were predominantly concentrated along the equator of the nanoparticle.

### 3.10. NDS-Filled Polymer Nanocomposites

Polymer nanocomposites, modified with NDS of various types, were obtained using two distinct types of polymer matrices and processing techniques. This was done to gain insight into the potential of NDS, distinguished by varying ratios of nanographite/ND phases, as a promising constituent HCNM for nanocomposite design.

**Figure 13:**
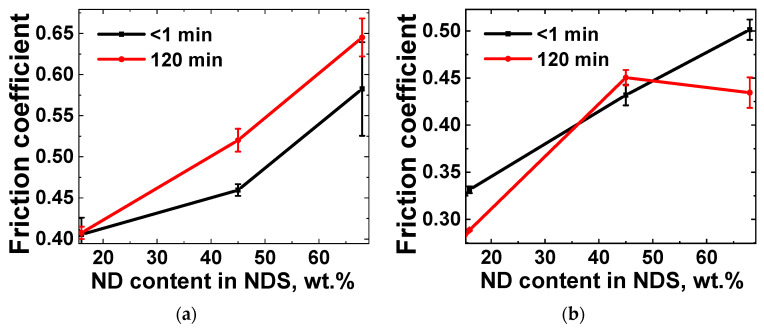
Dependencies of the friction coefficient on the ND content in the NDS powder (Table 2) in nanocomposites based on PP modified with (**a**) 2.5 and (**b**) 10 wt.% of NDS of various types. The curves were obtained for different durations (1 and 120 min) of annealing at 205 °C.

The tribological properties of the obtained PP-based nanocomposites were investigated with regard to the type and content of the NDS nanoparticles. Furthermore, the previously investigated effect of nanoparticle migration to the polymer composite melt boundaries during annealing at temperatures above the melting temperature of PP was studied [57]. The dependencies of the friction coefficient on time were obtained for PP-based nanocomposites containing 2.5 and 10 wt.% NDS particles of various types before and after annealing the composites at 205 °C for 2 h. The dependencies of the averaged friction coefficient values on the ND phase content in the NDS powder (see Table 2) for six independent runs are presented in Figure 13.

As observed for both selected values of the NDS content in the PP matrix (2.5 and 10 wt.%), an increase in the ND phase content in the NDS results in higher friction coefficient values (Figure 13). As previously outlined in the work [57], the annealing process results in the formation of a gradient structure within the nanocomposite material, provided that the filler content remains below the percolation threshold. This phenomenon is attributed to the migration of nanoparticles towards the boundaries of the polymer nanocomposite melt. These changes are reflected in the tribological properties of the nanocomposites, including the coefficient of friction, as well as the dissimilar characteristics of the composite surface and bulk. In particular, the results presented in Figure 13 demonstrate a direct correlation between the changes in the ND/nanographite ratio in the NDS particles and the impact of annealing on the friction coefficient of nanocomposite materials. Moreover, it can be observed that an increase in the bulk content of NDS from 2.5 wt.% to 10 wt.% results in a change to the dynamics of composite morphology evolution. In particular, the hybrid nature of the HCNM is revealed by the presence of a competing process of filler agglomeration. At higher NDS content values, the mobility of the ND particles is constrained, as they tend to form larger agglomerates. In contrast, the nanographite phase, which is less prone to agglomeration (due to the orientation of the nanoparticles, which prevents effective attachment), can more successfully form an enriched layer at the surface of the composites, effectively acting as a lubricant.

**Figure 14:**
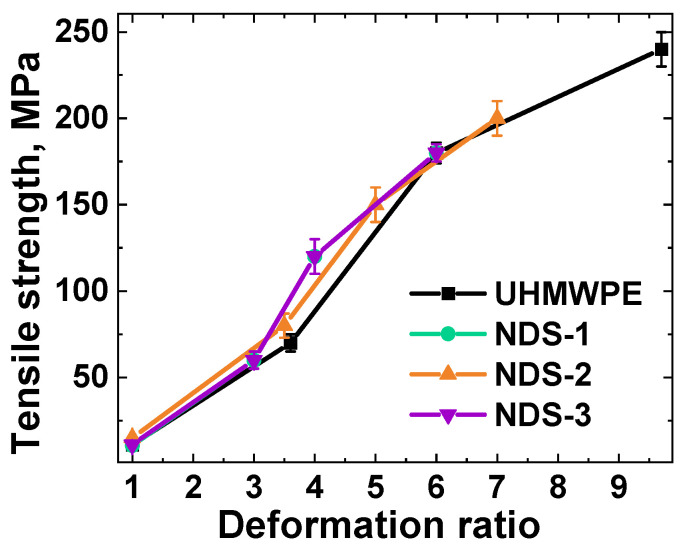
Dependence of the tensile strength on the homogeneous shear deformation ratio for the composites based on disentangled UHMWPE reactor powder and obtained using the solid-state processing approach without (“UHMWPE”) and with the addition of 10 wt.% NDS of various types.

The second nanocomposite system was obtained through the implementation of a solid-state processing procedure using disentangled UHMWPE and NDS particles of various types as the primary components. During the initial mixing of the components, the NDS particles attached to the RP nanoscale irregularities, including pores and protrusions, by mechanical means. Subsequently, the mixtures underwent the process of cold compaction. As a consequence of the solid-state nature of the processing, the NDS particles remained densely packed at the boundaries of the UHMWPE grains, thereby preventing their entry into the polymer grain volume. Moreover, the regions of contact between the NDS particles were devoid of any polymeric phase. This is regarded as an exemplar of the most extreme segregation of the filler within composites based on UHMWPE. Following pressure-induced compaction, such composite mixtures can be deformed under homogeneous shear conditions to obtain oriented samples, which exhibit higher tensile strength values [41]. In this case, NDS particles may potentially demonstrate their influence on the orientation strengthening of UHMWPE matrices. The results of the mechanical testing of materials based on disentangled UHMWPE RP obtained using the solid-state processing approach with and without the addition of 10 wt.% NDS of various types are presented in Figure 14.

As illustrated in Figure 14, the mechanical properties of the composites, modified with NDS particles with differing nanographite/ND ratios, exhibit a nearly linear and practically identical deformation behavior to that of the non-filled UHMWPE. This provides compelling evidence that the deformational behavior of the disentangled UHMWPE is not influenced by the presence of the filler during solid-state processing and orientation strengthening, even at high filler concentrations.

The findings for the two investigated polymer nanocomposite systems, with two distinct types of filler distribution—one close to uniform for nanocomposites based on PP and the other extremely segregated for nanocomposites based on UHMWPE—demonstrate that the NDS particles have the potential to serve as a modeling material. In particular, the properties of NDS-filled composite materials can be adjusted by varying the NDS explosive precursor while maintaining the filler content value. This could be effectively employed for the experimental verification of numerical, semi-empirical, and theoretical models of the relationships between composite structures, filler types, and filler contents. The obtained data could provide a solid foundation for the future development of new, versatile HCNM-based polymer nanocomposite materials.

## 4. Conclusions

The results presented in this study demonstrate that the NDS is a highly versatile HCNM. A variety of structural methods were employed to ascertain that NDS particles exhibit analogous morphological characteristics irrespective of the explosive precursor, with the sole distinction being the ratio of nanographite to ND phases. This allows for the accurate attribution of dynamic changes in the structure and properties of polymer nanocomposites during processing to the phase composition of the nanoparticles. In this regard, NDS can be employed as a model filler to investigate and subsequently predict the functional properties of composites that are specific to either the nanographite or ND phases, taking into account the well-described morphology of the NDS particles. The combination of the hard and chemically resistant ND phase and the electrically conductive nanographite in a single hybrid nanoparticle offers a promising avenue for the design of multi-purpose polymer nanocomposites for industrial-scale applications. This is particularly promising given the efficiency and low cost of detonation synthesis procedures.

## Figures and Tables

**Table 1:** Tribological test parameters.

Pin Material	AISI 420 (45HRc) Steel
Pin diameter, mm	5
Track radius, mm	25
Rotation speed, m/s	0.8
Test duration, h	8
Normal load, N	10

**Table 2:** ND and nanographite weight fraction values in the NDS of various types calculated using integration of the corresponding peaks in *I* − 2*θ* and *I*(*s*)*s*^2^*ds* − *s* coordinates (Figure 6) and nanographite domain size values obtained for 002 reflection using the Scherrer equation [51].

NDS Type	ND Fraction, wt.%		Nanographite Fraction, wt.%		Nanographite Domain Size (002 Reflection), nm
	*I*(*s*) − *s*	*I*(*s*)*∙s*^2^ − *s*	*I*(*s*) − *s*	*I*(*s*)*∙s*^2^ − *s*	
NDS-1	16	45	84	55	2.64
NDS-2	45	70	55	30	2.96
NDS-3	68	86	32	14	3.31

## Data Availability

The original contributions presented in this study are included in the article/Supplementary Material. Further inquiries can be directed to the corresponding author(s).

## Supplementary Materials

The following supporting information can be downloaded at: https://www.mdpi.com/article/10.3390/nano15010056/s1. Detonation Nanodiamond Soot—A Structurally Tailorable Hy-brid Graphite/Nanodiamond Carbon-Based Material: DLS and AFM Data (Supplementary Materials).
