# Supplementary material for: Detonation Nanodiamond Soot—A Structurally Tailorable Hybrid Graphite/Nanodiamond Carbon-Based Material
Source: Nanomaterials (Basel). 2025 Jan 1;15(1):56. doi: 10.3390/nano15010056 (PMC11722865; doi:10.3390/nano15010056)
Supplement: Supplementary file 1 [file nanomaterials-15-00056-s001.zip › nanomaterials-3346165-supplementary.pdf]

Detonation Nanodiamond Soot – A Structurally Tailorable Hybrid Graphite/Nanodiamond Carbon-Based Material: DLS and AFM Data (Supplementary Materials)

Tikhon S. Kurkin [1,2,*], Oleg V. Lebedev [1], Evgeny K. Golubev [1], Andrey K. Gatin [3], Victoria V. Nepomnyashchikh [1], Valery Yu. Dolmatov [4], and Alexander N. Ozerin [1]

[1] Enikolopov Institute of Synthetic Polymer Materials Russian Academy of Sciences~(ISPM RAS), Profsoyuznaya St. 70, 117393 Moscow, Russia; oleg.lebedev@phystech.su (O.V.L.); ozerin@ispm.ru (A.N.O.)

[2] Moscow Center for Advanced Studies, Kulakova Str. 20, 123592 Moscow, Russia

[3] N. N. Semenov Federal Research Center for Chemical Physics Russian Academy of Sciences (FRCCP RAS), Kosygina Street 4, 119991 Moscow, Russia

[4] Federal State Unitary Enterprise, Special Design and Technology Bureau 'Technolog', Sovetsky Prosp. 33a, 192076 Saint Petersburg, Russia

* Correspondence: kurkints@ispm.ru

The study of sedimentation stability and dispersibility of the NDS1, NDS-2, and NDS-3, which appear to be the most promising diamond-containing carbon nanomaterials for the purposes of further work in the direction of development, research and testing of new types of high-tech polymer nanocomposite materials for structural and functional purposes with special properties (high-strength, wear-resistant, with reduced flammability, with improved electrophysical characteristics), was carried out on the basis of the following systems. The compositions of the studied systems are presented in Table S1.

Table S1 - Compositions of the studied colloidal systems based on NDS.

NDS sample	Liquid media	NDS weight fraction, %
NDS-1	Water	0.5
	Hexane	0.5
	Acetone	0.5
	DMSO	0.5
	Ethanol	0.5
	Isopropanol	0.5
NDS-2	Water	0.5
	Hexane	0.5
	Acetone	0.5
	DMSO	0.5
	Ethanol	0.5
	Isopropanol	0.5
NDS-3	Water	0.5
	Hexane	0.5
	Acetone	0.5
	DMSO	0.5
	Ethanol	0.5
	Isopropanol	0.5

The colloidal systems listed in Table S1 were prepared according to the following general scheme: a suspension of NDS was redispersed in liquid dispersion medium at room temperature, after which the obtained dispersion of 25 ml was subjected to processing on a laboratory ultrasonic disperser CUD-500 manufactured by Criamid (Russia, Moscow) in adiabatic mode with an exposure time of 60 s.

To obtain information on the size distribution of NDS aggregate/agglomerate in the studied colloidal systems the method of electrophoretic dynamic light scattering (Doppler light beat spectroscopy) was used. Measurements were performed on a laser (λ=750 nm) analyzer Zetatrac (Microtrac, Inc.), by the method of "reflection" imaging (the angle of scattered signal registration is 180° with respect to the direction of the primary beam). The range of sizes (hydrodynamic diameters) of scattering NDS aggregates/agglomerates registered by the device according to the manufacturer's data: from 0.001 μm to 6.500 μm, therefore it was not possible to obtain information about the NDS aggregate/agglomerate size distribution of the lower dispersed fraction (or qualitatively establish its presence or absence). Signal processing and calculations were carried out in the approximation of optically opaque nonspherical particles for all investigated systems.

Figures S1-S4 show the numerical distributions of NDS-1 aggregates/agglomerates in acetone (Figure S1), DMSO (Figure S2), ethanol (Figure S3) and isopropanol (Figure S4).

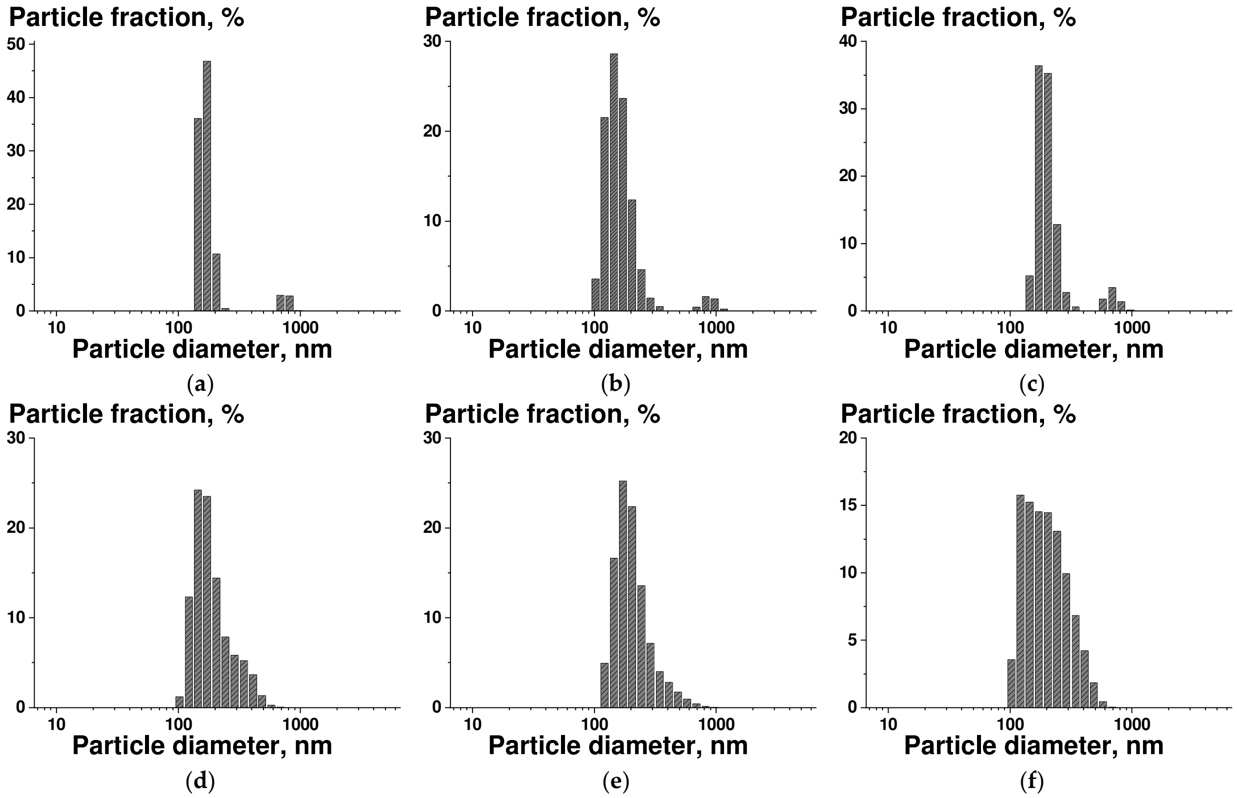

Figure S1 - Numerical size distributions of NDS-1 aggregates/agglomerates in acetone according to dynamic light scattering data: (a) - immediately after ultrasonic treatment; (b) - after 24 hours; (c) - after 120 hours; (d) - after 1 week; (e) - after 3 weeks; (f) - after 4 weeks.

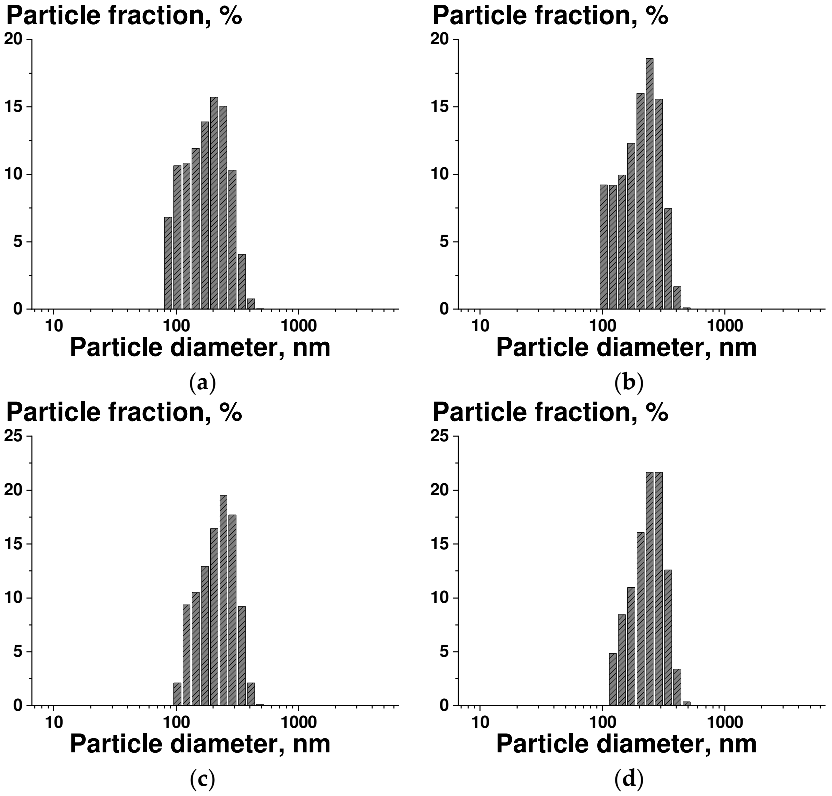

Figure S2 - Numerical size distributions of NDS-1 aggregates/agglomerates in DMSO according to dynamic light scattering data: (a) - immediately after ultrasonic treatment; (b) - after 24 hours; (c) - after 120 hours; (d) - after 1 week.

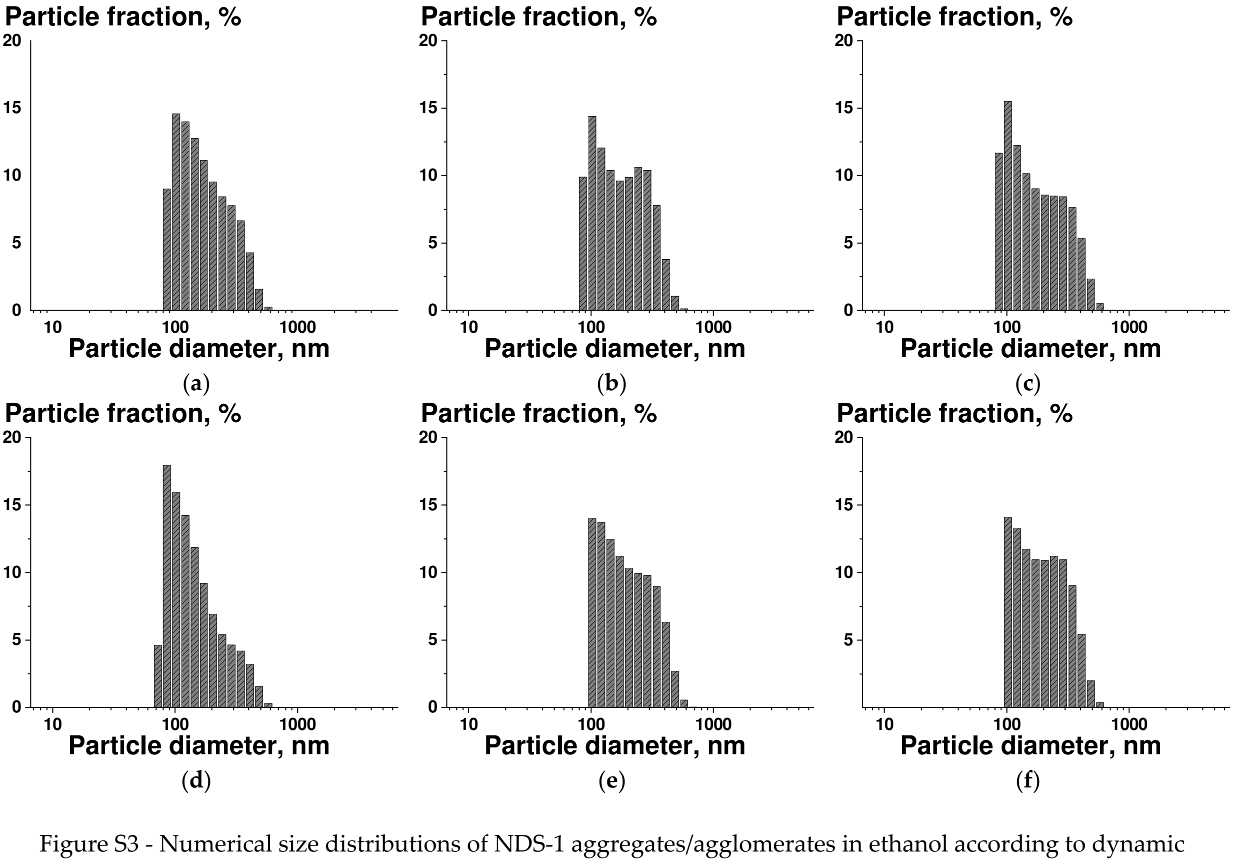

Figure S3 - Numerical size distributions of NDS-1 aggregates/agglomerates in ethanol according to dynamic light scattering data: (a) - immediately after ultrasonic treatment; (b) - after 24 hours; (c) - after 120 hours; (d) - after 1 week; (e) - after 3 weeks; (f) - after 4 weeks.

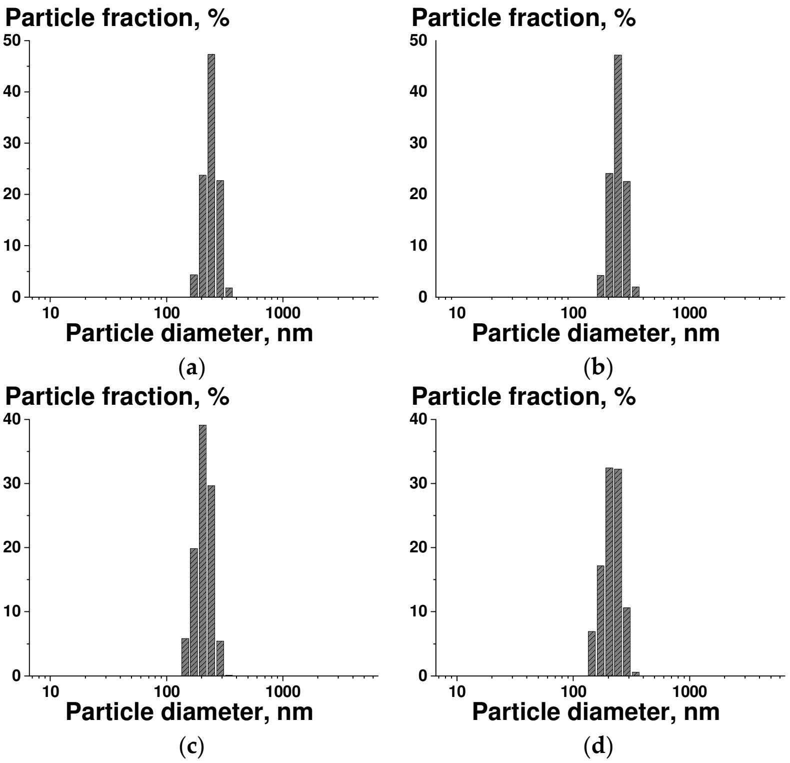

Figure S4 - Numerical size distributions of NDS-1 aggregates/agglomerates in isopropanol according to dynamic light scattering data: (a) - immediately after ultrasonic treatment; (b) - after 24 hours; (c) - after 120 hours; (d) - after 1 week.

The numerical distributions of NDS-2 aggregates/agglomerates in DMSO (Figure S5), ethanol (Figure S6) and isopropanol (Figure S7) are presented below. There is no sedimentation stability in acetone (complete sedimentation time is 85 seconds).

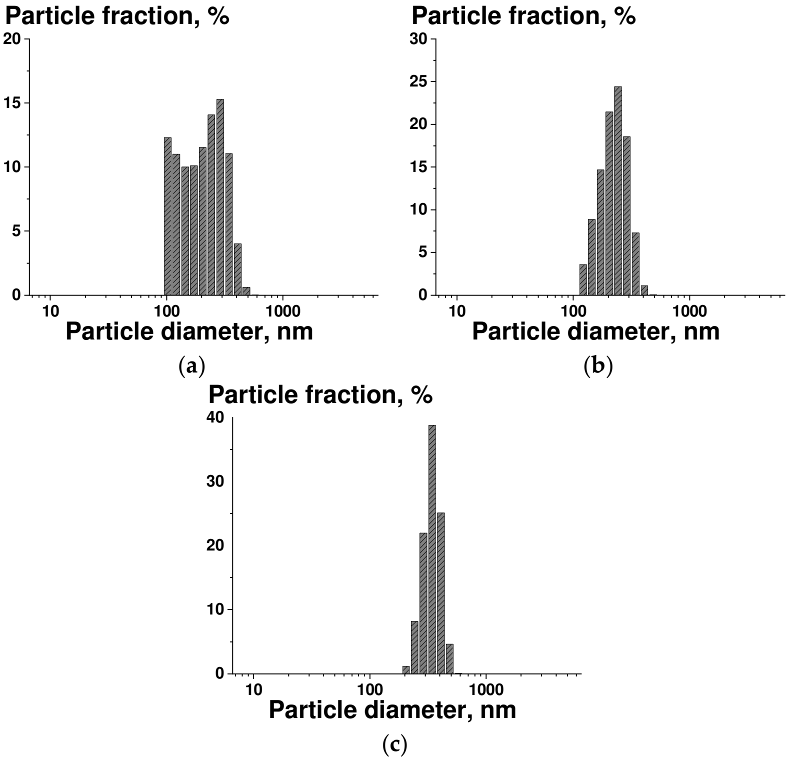

Figure S5 - Numerical size distributions of NDS-2 aggregates/agglomerates in DMSO according to dynamic light scattering data: (a) - immediately after ultrasonic treatment; (b) - after 120 hours; (c) - after 1 week.

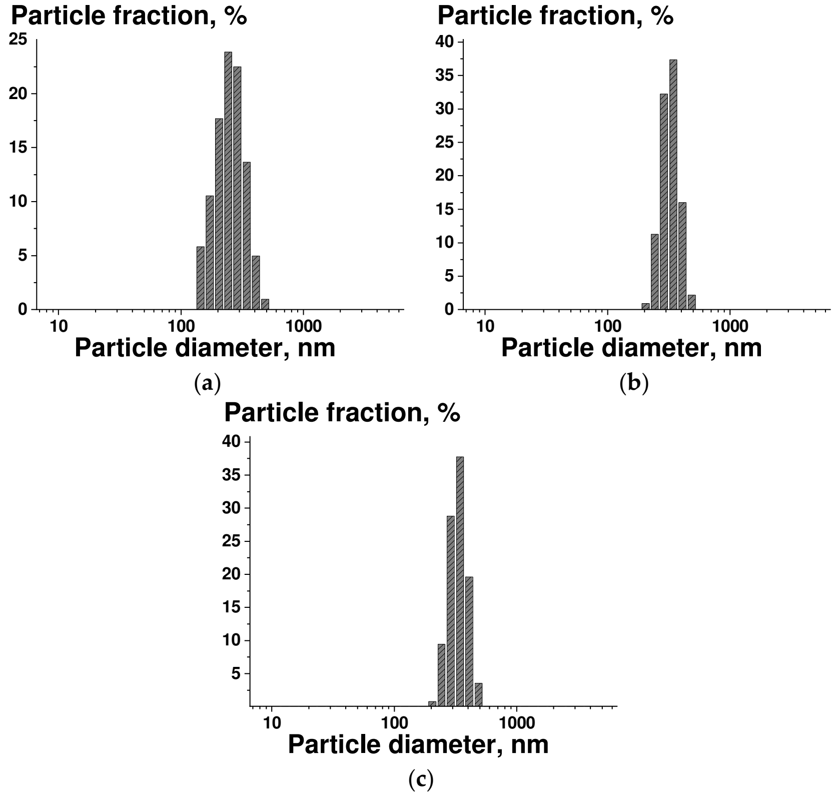

Figure S6 - Numerical size distributions of NDS-2 aggregates/agglomerates in ethanol according to dynamic light scattering data: (a) - immediately after ultrasonic treatment; (b) - after 5 hours; (c) - after 24 hours. After 48 hours the system appears to be completely sedimented.

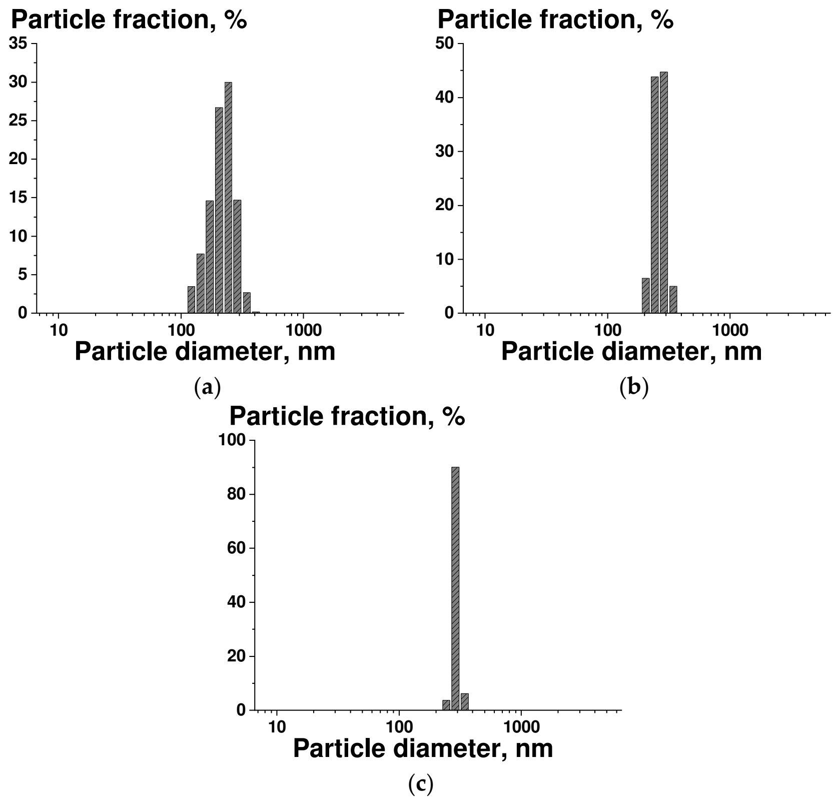

Figure S7 - Numerical size distributions of NDS-2 aggregates/agglomerates in isopropanol according to dynamic light scattering data: (a) - immediately after ultrasonic treatment; (b) - after 120 hours; (c) - after 1 week.

The numerical distributions of NDS-3 aggregates/agglomerates in acetone (Figure S8), DMSO (Figure S9), ethanol (Figure S10) and isopropanol (Figure S11) are presented below.

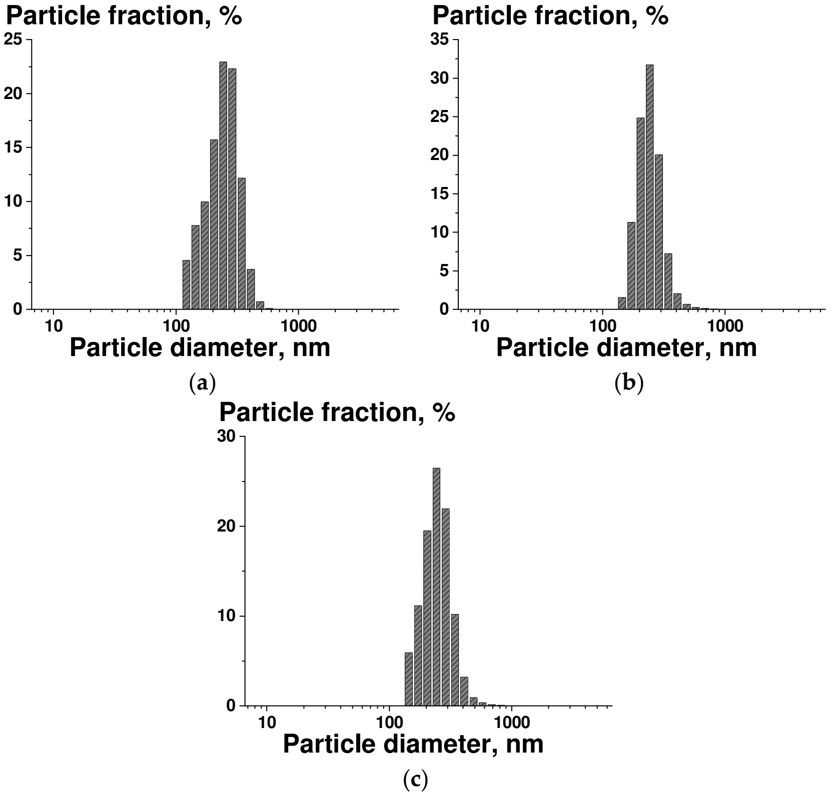

Figure S8 - Numerical size distributions of NDS-3 aggregates/agglomerates in acetone according to dynamic light scattering data: (a) - immediately after ultrasonic treatment; (b) - after 120 hours; (c) - after 1 week.

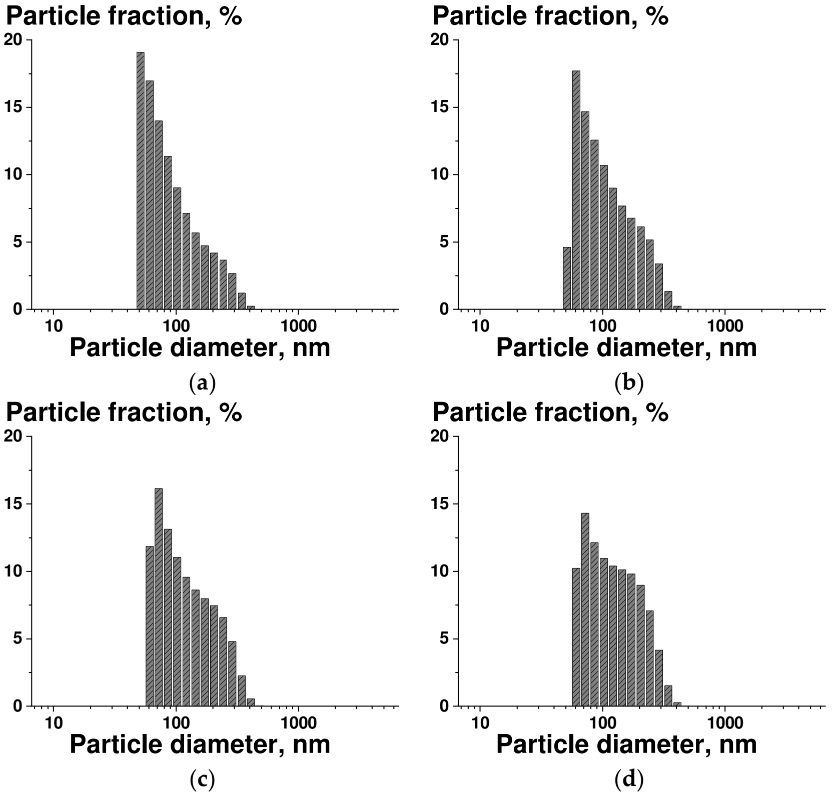

Figure S9 - Numerical size distributions of NDS-3 aggregates/agglomerates in DMSO according to dynamic light scattering data: (a) - immediately after ultrasonic treatment; (b) - after 24 hours; (c) - after 120 hours; (d) - after 1 week.

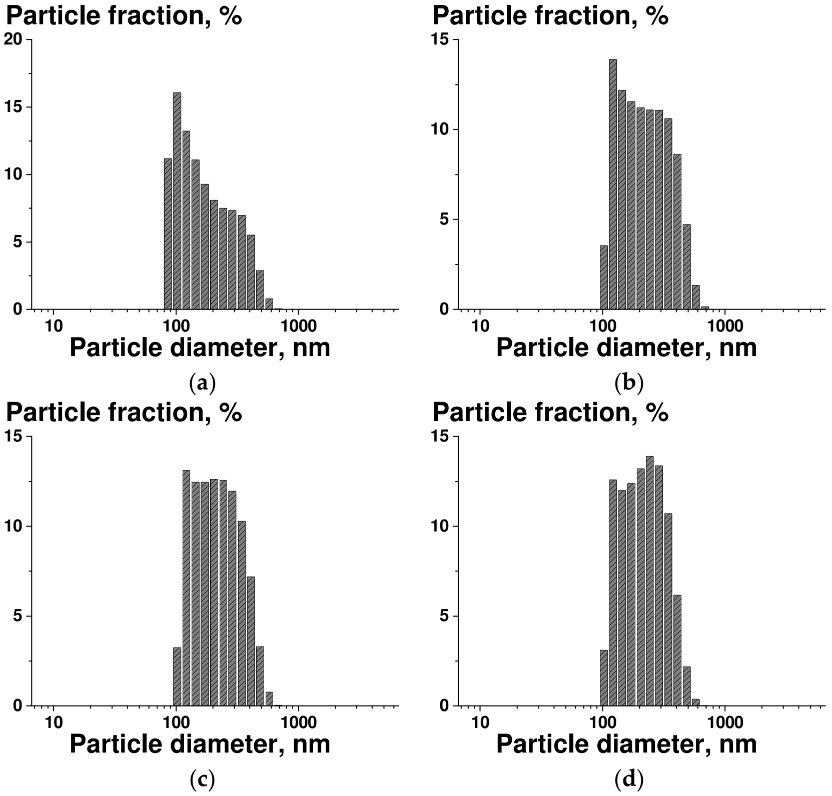

Figure S10 - Numerical size distributions of NDS-3 aggregates/agglomerates in ethanol according to dynamic light scattering data: (a) - immediately after ultrasonic treatment; (b) - after 24 hours; (c) - after 120 hours; (d) - after 1 week.

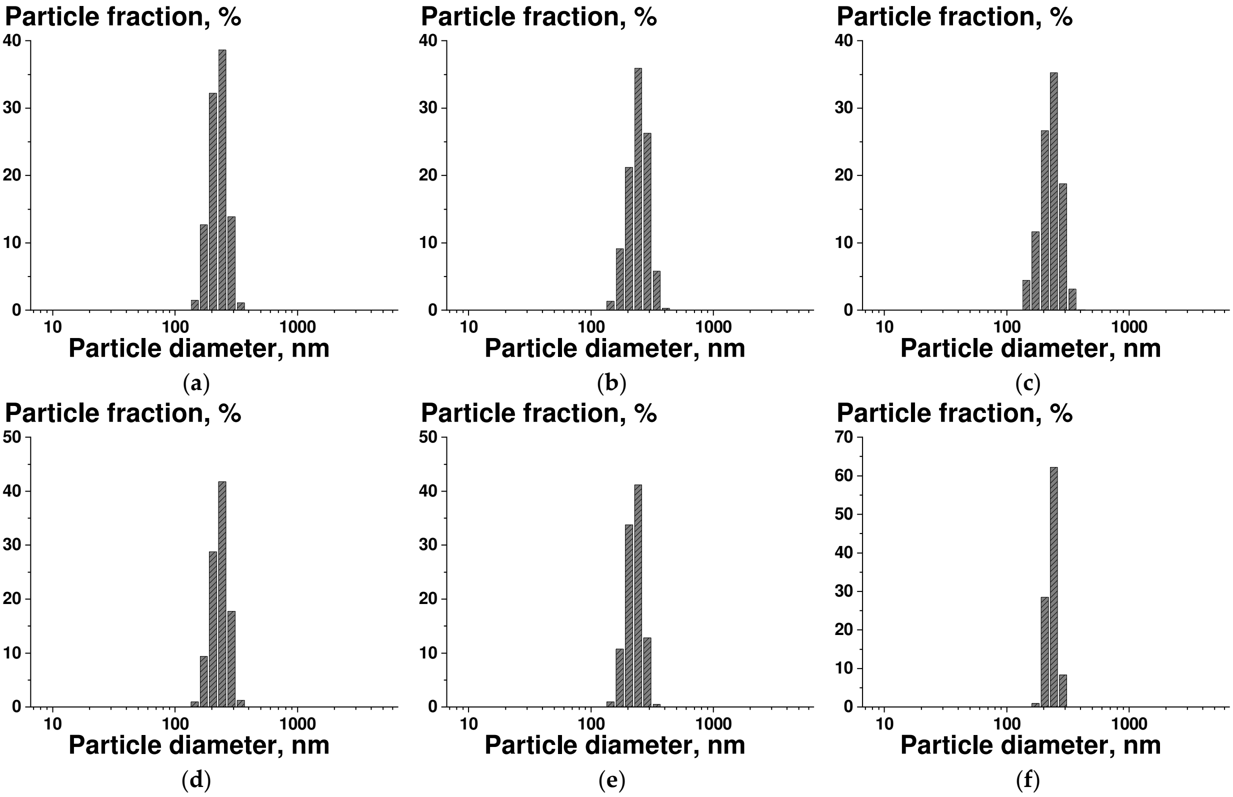

Figure S11 - Numerical size distributions of NDS-3 aggregates/agglomerates in isopropanol according to dynamic light scattering data: (a) - immediately after ultrasonic treatment; (b) - after 24 hours; (c) - after 120 hours; (d) - after 1 week; (e) - after 3 weeks; (f) - after 4 weeks.

In the Figure S12 the photograph of the NDS-1 dispersion in acetone after one year is presented.

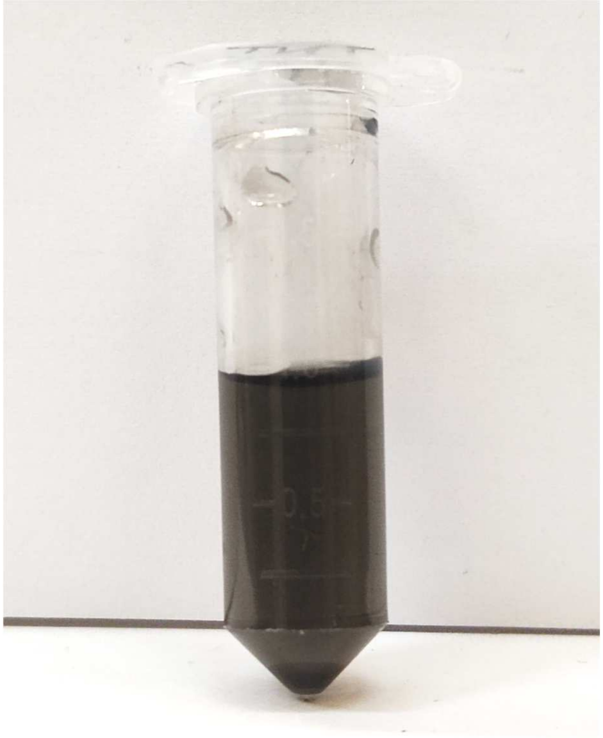

Figure S12 - Photograph of the NDS-1 dispersion in acetone after one year.

AFM was used as another method for visualization of NDS agglomerate size distribution and NDS particle morphology. The results of the AFM studies of the NDS powders of different types are presented in Figure S13. AFM measurements were performed using a Solver HV atomic force microscope (NT-MDT, Moscow, Russia). Standard HA-NC cantilevers were used for the measurements. The dimensions of the chip were 3.6 × 1.6 × 0.45 mm, while the dimensions of the cantilever were 87 × 32 × 1.75 μm, with a curvature radius of the tip of 10 nm and a force constant of 5.8 N/m. The measurements were conducted in tapping mode, with the acquisition of surface topography and phase contrast. Prior to the AFM investigations, NDS dispersions in isopropanol (with a solid phase concentration of 0.5 wt.%) were deposited on a highly oriented pyrolytic graphite substrate and allowed to dry in air at room temperature.

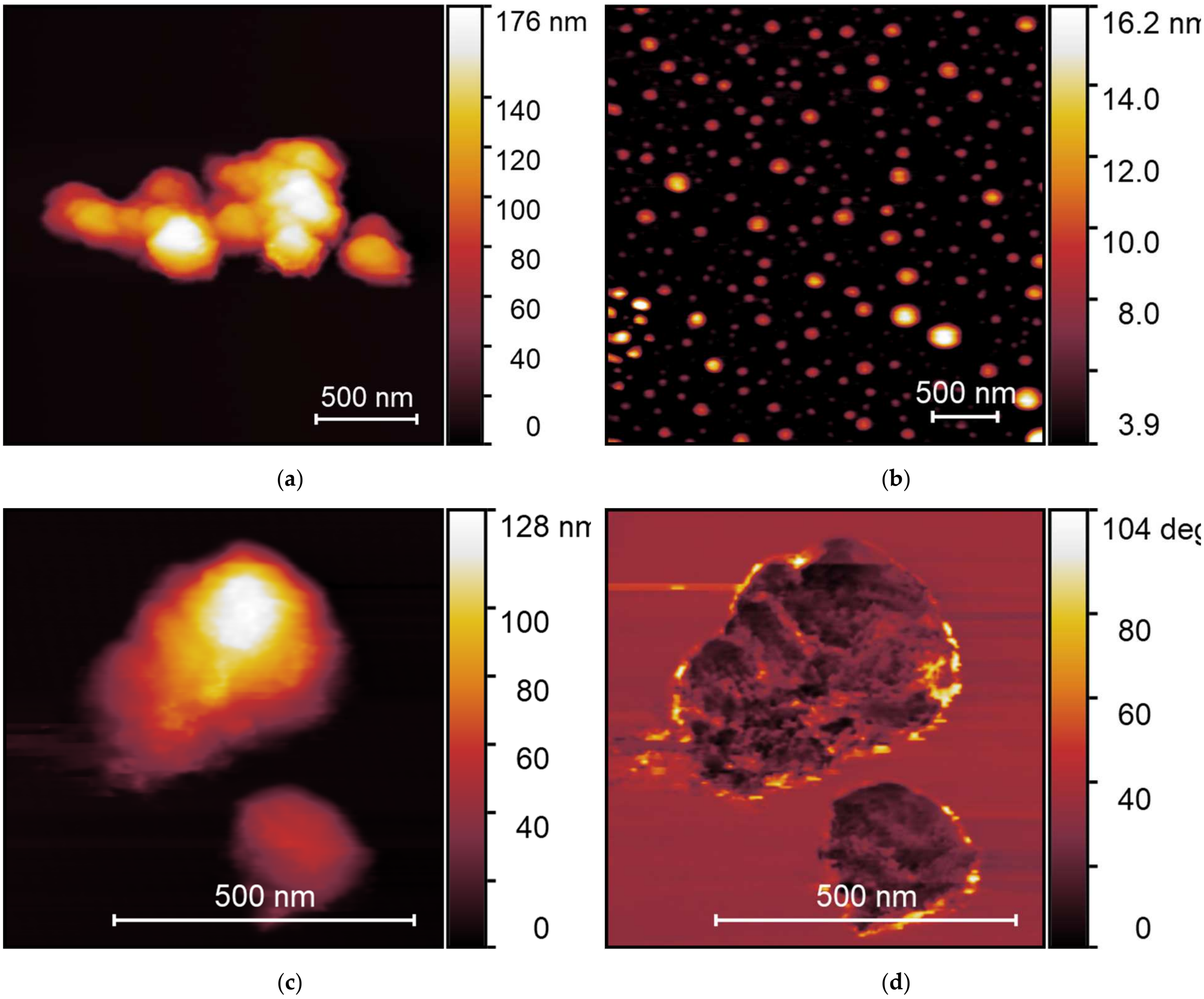

Figure S13 - (**a**,**b**,**c**) Height distribution; and (**d**) phase contrast mode images obtained for the (**a**) NDS-1, (**b**) NDS-2, and (**c**,**d**) NDS-3 powders using AFM in the semi-contact mode.

It can be seen in Figure S13a that a large NDS-1 particles agglomerate with diameter of ~1.5 µm comprises small strong-coupled [1] aggregates with diameter of ~250 nm and thickness of ~50 nm. Profiling analysis of AFM topographic images for NDS-2 powders revealed that the NDS-2 aggregate dimensions in average are ~170 nm with a thickness of only ~15 nm. For diamond-enriched NDS-3 powder the aggregates turned out to be anisometric with diameter ~250 nm and thickness of ~50 nm. The obtained AFM results correlate with the SEM, DLS, and LDA data.

Reference

1. Schaefer, D.W.; Justice, R.S. How Nano Are Nanocomposites? *Macromolecules* **2007**, *40*, 8501–8517, doi:10.1021/ma070356w.
